# Burnout and Engagement Within Medical Education: A Repeated Measures Study on Their Evolution and Main Determinants

**DOI:** 10.5334/pme.2013

**Published:** 2026-02-16

**Authors:** Anke Boone, Jonas Steel, Olivia Lavreysen, Marie-Claire Lambrechts, Sofie Vandenbroeck, Lutgart Braeckman, Nele Michels, Dirk Devroey, Ann Roex, Hanne Kindermans, Lode Godderis

**Affiliations:** 1 Centre for Environment and Health, Department of Public Health and Primary Care, University of Leuven, Leuven, Belgium; 2 IDEWE, External Service for Prevention and Protection at Work, Leuven, Belgium; 3 VAD, Flemish Centre of Expertise on Alcohol and Other Drugs, Brussels, Belgium; 4 Department of Public Health and Primary Care, Faculty of Medicine and Health Sciences, Ghent University, Ghent 9000, Belgium; 5 EURACT & Centre for General Practice Training (ICHO), Flanders, Belgium; 6 Department of Family Medicine and Chronic Care, Faculty of Medicine and Pharmacy, Vrije Universiteit Brussel, Brussels, Belgium; 7 Department of Clinical Sciences, Faculty of Medicine and Pharmacy, Vrije Universiteit Brussel, Brussels, Belgium; 8 Research Group Healthcare and Ethics, Faculty of Medicine and Life Sciences, Hasselt University, Diepenbeek, Belgium

## Abstract

**Introduction:**

Burnout is a major concern in medical education and healthcare. Despite growing attention, little is known about how burnout and engagement evolve within the different phases of medical education or which factors shape these outcomes within each phase. This study assesses phase-specific trajectories of burnout and engagement and identifies the demands, resources, and personality traits associated with these outcomes.

**Methods:**

A prospective cohort study with three annual measurement waves was conducted among medical students and residents (n = 1.257) from all five universities in Flanders, Belgium. Three cohorts (i.e. bachelor students, master students, and residents) were followed for three consecutive years, using an open cohort design. Data were collected annually: at baseline (T0), after one year (T1), and after two years (T2). Linear mixed-effects models and cross-lagged panel analyses were used to examine temporal changes in burnout complaints and engagement; and to identify key demands, resources and personality traits within each cohort.

**Results:**

Within each cohort, burnout complaints increased gradually over time, while engagement declined. Within all learning phases, workload and work-home conflict were identified as primary demands, whereas meaningfulness was identified as the main resource, followed by learning opportunities. Neuroticism was positively associated with burnout complaints and negatively with engagement, while perfectionistic strivings correlated positively with engagement.

**Conclusion:**

This study demonstrates how burnout complaints and engagement evolve within learning phases in medical education and identifies key determinants underlying these trajectories. Efforts should prioritize reducing perceived workload and work–home conflict, while strengthening meaningfulness and learning opportunities. In addition, acknowledging individual personality traits may help tailor interventions.

## Introduction

Burnout represents a major concern in demanding work environments such as healthcare [1,2,3,4,5]. It is commonly described as a response to chronic work-related stress, characterized by three complaints: emotional exhaustion, cynicism, and reduced professional efficacy [5,6,7]. Engagement, the counterpart to burnout, reflects a positive mental state in which individuals feel energized and deeply immersed in their work [8]. Both burnout and engagement in healthcare are relevant subjects of study because of their individual, organizational and societal impacts, including associations with career choice regret, patient satisfaction and quality of care [9,10,11].

Within the healthcare context, medical students and residents appear particularly vulnerable to burnout [2,4,12]. Previous studies report high prevalence rates, with burnout estimated at 37.2% among medical students (95% CI, 32.66–42.05%) and 47.3% among residents (95% CI, 43.1–51.5%) [2,4]. Moreover, burnout levels in both groups exceed those observed in the general population (p < .001) [12]. In contrast, studies on engagement indicate that residents report lower engagement levels than more senior physicians [13].

Despite increasing attention to burnout and engagement, evidence on how burnout and engagement evolve within the course of medical education remains scarce, particularly from repeated-measures designs [14,15,16]. Furthermore, prior research on burnout trajectories within medical education has typically examined (undergraduate) medical students or residents, rather than integrating multiple stages of medical training [14,17,18]. With regard to engagement, to our knowledge, only one study conducted in China has systematically examined the evolution of engagement across successive phases of medical education; however, this study was limited to undergraduate medical training [19].

To address these gaps, the present study investigates temporal patterns of burnout complaints and engagement within different phases of medical education. Based on previous findings, we hypothesize a progressive increase in burnout complaints over the course of training, accompanied by a corresponding declining trend in engagement (Hypothesis 1a) [13,14,17,18,19]. Additionally, we explore differences in burnout complaints between general practice (GP) residents and residents in other specialities, given meta-analytic evidence indicating lower burnout prevalence in GP medicine compared to hospital-based specialties, such as radiology and surgery (Hypothesis 1b) [20].

Beyond investigating the evolution of burnout complaints and engagement within medical education phases, a second objective of the present study is to identify key demands and resources that most strongly influence burnout complaints and engagement within these different phases. Prior evidence suggests that the nature of demands may shift within the educational continuum [14]. For instance, early phases of medical education are predominantly characterized by challenges common to university entry and a heavy academic workload, while later phases of training introduce different demands, such as increased clinical responsibility [14].

To address the second research objective, this study adopts the Job Demands-Resources (JD-R) model as its guiding theoretical framework [21,22]. The JD-R model is a well-established and empirically supported framework that accommodates a wide range of demands and resources, and explains their influence on both burnout and engagement [21,22,23]. Within this framework, job demands are defined as physical, psychological, social, or organizational aspects of work that incur sustained costs, while job resources comprise aspects of the job that facilitate goal attainment, mitigate demands, and stimulate personal growth [22]. Recent extensions of the JD-R framework incorporate personality traits as factors that may increase vulnerability to burnout and enhance engagement [24,25].

Although the JD-R model is highly applicable to residents given their clinical work environment, adaptations are required to adequately capture the demands and resources encountered by medical students during preclinical training. This has resulted in the development of the Study Demands-Resources (SD-R) model, which translates the JD-R framework to educational settings [26,27]. The SD-R framework has been successfully validated in educational contexts, including medical education, where burnout and engagement are conceptualized as outcomes of the interaction between study-related demands and resources [27,28,29,30,31].

**Figure 1:**
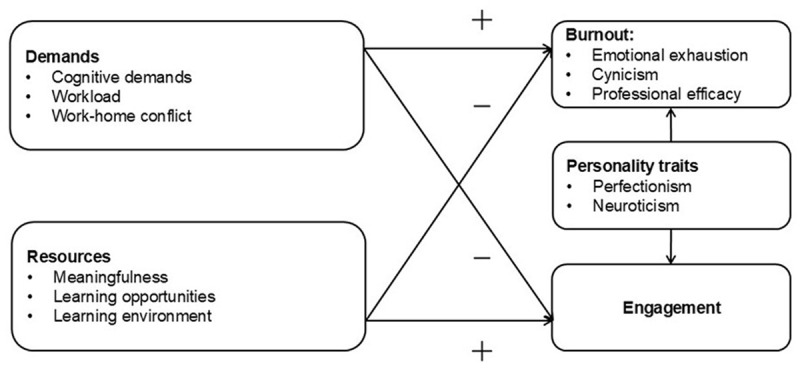
Study Demands-Resources (SD-R) Model.

Among the various demands and resources linked to burnout and engagement, this study focuses on those with the strongest empirical support for all learning phases of medical training. The primary demands identified include cognitive demands, workload, and work-home conflict, while key resources encompass meaningfulness, learning opportunities, and a supportive learning environment [14,32,33,34,35,36]. Additionally, neuroticism and perfectionism have been identified as relevant vulnerability factors [25,37,38]. Specifically, perfectionistic concerns, such as fear of making mistakes, are associated with higher burnout levels, whereas perfectionistic strivings, characterized by high personal standards, tend to correlate with lower burnout [37,38]. Figure 1 illustrates the relationships between demands, resources, personality traits, burnout, and engagement as derived from prior empirical research.

Despite growing attention to the demands and resources driving burnout and engagement in medical education, evidence remains limited on which factors are most influential within specific learning phases, hindering the development of phase-specific interventions. Based on prior research, we hypothesize that cognitive demands and a heavy academic workload will have a strong impact during the preclinical (early) years, whereas cognitive demands, workload and work–life conflict are expected to be significant during the clinical (later) phases (Hypothesis 2a) [14,32,33,34,39,40,41]. Regarding key resources, we hypothesize that the learning environment is the main protective resource of burnout and engagement within all phases of medical education, followed by learning opportunities, with meaningfulness expected to be particularly influential during residency due to increased clinical responsibility and direct patient care (Hypothesis 2b) [29,36,39,42,43,44,45].

## Methods

### Study Design and Ethics Approval

This study is part of the WeMeds research project (www.wemeds.be) and received approval from the Ethics Committee Research UZ/KU Leuven in April 2021 (S64150). All participants provided signed informed consent prior to enrollment. Participation was voluntary, uncompensated, and conducted in accordance with the Declaration of Helsinki’s ethical principles for medical research involving human subjects. The present study focuses on the quantitative part of the Wemeds study, namely a prospective cohort design with three repeated measures collected via a self-administered online survey.

### Population and Recruitment

Medical students and residents from all five medical faculties in Flanders (Belgium) – University of Leuven (KU Leuven), Free University of Brussels (VUB), University of Hasselt (UH), University of Ghent (UG), and University of Antwerp (UA) – were invited to participate. Eligible participants were Dutch-speaking, at least 18 years old, and enrolled as first-year bachelor students (cohort 1), first-year master students (cohort 2), or first-year residents (cohort 3) at T0. In Belgium, first-year bachelor students are equivalent to first-year undergraduates, while first-year master students are fourth-year medical students with more specialized knowledge. Residents were divided into two groups based on their professional setting: GP residents, working in GP settings, and other residents, primarily based in hospitals.

The survey was administered annually over three consecutive academic years: 2021–2022 (T0, baseline), 2022–2023 (T1), and 2023–2024 (T2). Recruitment utilized both direct and indirect online channels, including emails, the WeMeds website, event flyers, and various social media platforms. The recruitment period ran from October to February each year, with monthly reminders (i.e. four in total) sent to the universities and relevant organisations. Interested individuals could register online immediately upon invitation, enabling survey completion and automatic notification for subsequent measurement moments. An open cohort design was implemented, allowing registered participants to be invited annually for follow-up measurements, while also permitting the enrollment of new participants at each time point.

### Data Collection

#### Burnout

For students, “burnout” was measured via three burnout dimensions (i.e., emotional exhaustion, cynicism, professional efficacy), using the Dutch version of the 15-item “Maslach Burnout Inventory – Student Survey (MBI-SS)” [46,47]. The internal consistency, measured by Cronbach’s alpha, was 0.89 for emotional exhaustion, 0.85 for cynicism, and 0.76 for professional efficacy. For residents, the 20-item Dutch version of the MBI for contact-based professions was used: “Utrecht Burnout Scale – Contact-based professions (UBOS-C)” [48]. Emotional exhaustion achieved a Cronbach’s alpha of 0.91, cynicism of 0.76 and professional efficacy of 0.83.

#### Engagement

“Engagement” was measured among medical students and residents, respectively with the 9-item Dutch ‘student’ version and the original version of the “Utrecht Work Engagement Scale (UWES)” [49,50]. Respondents rated the frequency of their experiences on burnout complaints and engagement using a 7-point Likert scale, ranging from “never” (0) to “every day” [6]. The Cronbach’s alpha was high for both groups: for students it reached 0.90 and for residents 0.91.

#### Demands

“Cognitive demands” (4 items), “workload” (4 items), and “work-home conflict” (4 items) were measured using corresponding scales from the Dutch “Copenhagen Psychosocial Questionnaire III” (COPSOQ; Burr et al., 2019). The internal consistency, measured by Cronbach’s alpha, was 0.62 for cognitive demands, 0.80 for workload, and 0.91 for work-home conflict.

#### Resources

“Meaningfulness” (2 items), and “learning opportunities” (3 items) were measured using corresponding scales from the Dutch “Copenhagen Psychosocial Questionnaire III” (COPSOQ; Burr et al., 2019). Cronbach’s alpha for meaningfulness was 0.83, while for learning opportunities this was 0.74. The “learning environment” (2 items) was measured using the corresponding scales of the “Dutch Residency Educational Climate Test” [51]. Items were measured on a 5-point Likert scale from “never” (0) to “always” (4) [52]. Cronbach’s alpha was 0.78.

#### Personality Traits

“Perfectionism” (8 items) was assessed using the “Frost Multidimensional Perfectionism Scale – Brief (FMPS-B)” [53], which distinguishes between perfectionistic strivings (4 items) and perfectionistic concerns (4 items). For perfectionistic strivings, Cronbach’s alpha was 0.87 and for perfectionistic concerns this was 0.78. “Neuroticism” (3 items) was measured with the corresponding scale from the “Big Five Inventory-Extra Short Form” (BFI-XS) [54]. Responses were rated on a 5-point Likert scale from “totally disagree” (1) to “totally agree” (5). Cronbach’s alpha for neuroticism was 0.77. The Dutch versions of these scales were obtained via back-translation, whereby the target-language versions were translated back into the source language by two independent translators from an external institute to verify accuracy [55].

### Statistical Analysis

All analyses and visualizations were conducted using R, version 4.3.0 [56]. Missing data resulted from participant drop-out and the open-cohort design. At the item level, when less than 5% of responses were missing, mean imputation was applied to retain usable cases, consistent with recommended practice for small proportions of missing data [14,57]. If more than 5% of an item was missing, the item was omitted. Linear mixed-effects models were then estimated using maximum likelihood estimation, which appropriately accommodates remaining missing outcomes data [14].

Data were analyzed as two subsets: a full dataset (participants with ≥1 time point) and a complete dataset (participants with all 3 time points). The full dataset maximized statistical power, while the complete dataset was used for robustness checks. To test whether data were Missing Completely At Random (MCAR), t-tests compared participants who responded at 2 time points with those who responded only once. Descriptive statistics were calculated for both datasets, including means, standard deviations, and Cronbach’s alpha for reliability. Normality was assessed using histograms, Q-Q plots, skewness, and kurtosis [58]. Pearson’s correlations (p < .05; r ≥ .30 considered meaningful) were used to examine relationships between variables [59].

LMMs were used to model changes in emotional exhaustion, cynicism, professional efficacy, and engagement within learning phases (Hypothesis 1a, b), accounting for data clustering. Separate LMMs were estimated for each outcome and for each cohort (bachelor students, master students, and residents). Fixed effects included cohort, measurement year, gender, and university; random effects included participant ID and measurement year. Differences between GP and other residents were assessed using t-tests with Benjamini-Hochberg correction. Additional LMMs tested Hypotheses 2a,b, assessing associations between demands, resources, personality traits, and outcomes, including multiple fixed effects (measurement year, cohort, gender, university, demands, resources, and personal traits) and random effects (participant ID and measurement year). Independent variables were centered and scaled for interpretability. Cross-lagged panel analyses examined whether variables at T0 predicted outcomes at T1, and T1 predicted T2 outcomes. These analyses were conducted on the full dataset to ensure power. Statistical significance was set at p < .05 with Benjamini-Hochberg correction for multiple comparisons.

## Results

### Sociodemographic Information of Participants and Descriptives

In total, 790 participants completed 1257 responses in the full dataset, resulting in an overall response rate of 14%. The mean age of the participants was 22.46 (SD = 3.96), 72.15% were female (N = 917), and 96.87% (N = 1.227) had no children. Of all participants, 465 participants completed one measurement only, 183 participated twice, and 142 three times. T-tests showed no significant differences between participants with one versus two measurements, suggesting random attrition (Supplementary Table S.1). Furthermore, the highest item-level missingness was 2.35%, which was below the commonly accepted threshold of 5%.

Although the response rate was relatively low, the composition of our sample closely reflects that of the target population. Female participants constituted the majority of our sample, which aligns with their high representation within the medical student and resident population. For residents, official numbers from the *Annual Statistics on Health Care Practitioners 2024* of the Federal Public Service Health, Food Chain Safety and Environment, indicate that women comprise 68% of GP residents and 60% of other residents [60]. For medical students, data provided by the five participating faculties indicated that, on average, 67% are female.

In addition, Supplementary Table S.2 shows the participant characteristics of the full dataset. In this dataset, cohort 3 included 151 GP residents and 174 other residents. Similar participant characteristics of the complete dataset can be found in Supplementary Table S.3, while Supplementary Table S.4 details GP residents and other residents in full and complete datasets. Supplementary Table S.5 provides the outcome variables per cohort in the full dataset, including means, standard deviations, score ranges, skewness, kurtosis and Cronbach’s alpha. The same details for the complete dataset are in Supplementary Table S.6. Additionally, correlation tables, presenting Pearson correlation plots for all study variables per cohort, are shown in Supplementary Table S.7.

### Evolution of Burnout Complaints and Engagement

**Figure 2:**
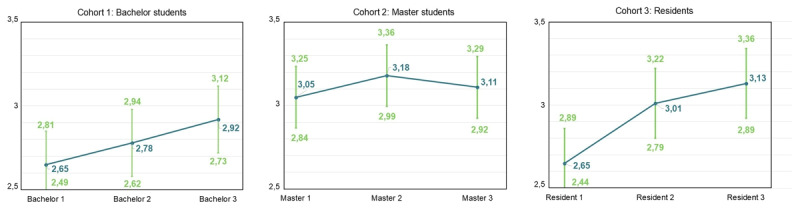
Predicted means pattern for each cohort for emotional exhaustion (full dataset).

LMMs were used to examine changes within the three cohorts for emotional exhaustion, cynicism, professional efficacy, and engagement (Hypothesis 1a), and to compare GP residents with other residents (Hypothesis 1b). The reference groups were first-year bachelor students, first-year master students and first-year residents. For emotional exhaustion, results indicate a significant increase in bachelor year 3 (β = 0.267, SE = 0.105, p < .05), resident year 2 (β = 0.345, SE = 0.116, p < .01) and resident year 3 (β = 0.468, SE = 0.135, p < .01) (Supplementary Table S.8). Figure 2 displays the predicted means. The t-test results with Benjamini-Hochberg correction show differences between GP and other residents, though none are statistically significant (Supplementary Table S.9, Supplementary Figure S.1).

**Figure 3:**
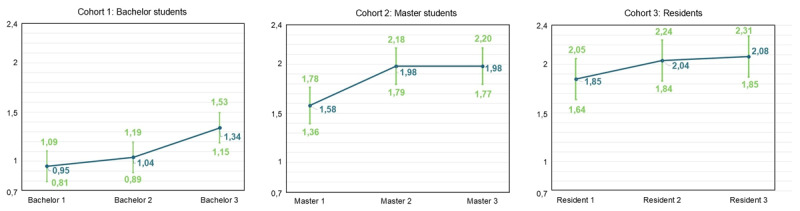
Predicted means pattern for each cohort for cynicism (full dataset).

For cynicism, results indicate a significant increase in bachelor year 3 (β = 0.394, SE = 0.108, p < .001), master year 2 (β = 0.408, SE = 0.094, p < .001) and master year 3 (β = 0.409, SE = 0.133, p < .01) (Supplementary Table S.10). Figure 3 displays the predicted means pattern. T-test results with Benjamini-Hochberg correction showed a statistically significant difference between GP residents year 2 and other residents in year 2 (β = 0.528, SE = 0.199, p < .05) (Supplementary Table S.11, Supplementary Figure S.2).

**Figure 4:**
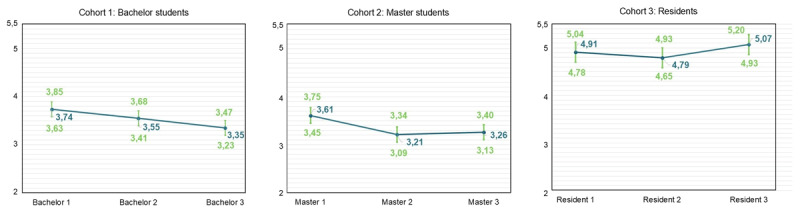
Predicted means pattern for each cohort for professional efficacy (full dataset).

For professional efficacy, results indicate a significant decrease in bachelor year 2 (β = –0.195, SE = 0.072, p < .01), bachelor year 3 (β = –0.391, SE = 0.076, p < .001), master year 2 (β = –0.388, SE = 0.072, p < .001) and master year 3 (β = –0.343, SE = 0.089, p < .001) (Supplementary Table S.12). Figure 4 displays the predicted means pattern. The t-tests with Benjamini-Hochberg correction show a significant difference between GP residents year 3 and other residents year 3 (β = –0.360, p < .05) (Supplementary Table S.13, Supplementary Figure S.3).

**Figure 5:**
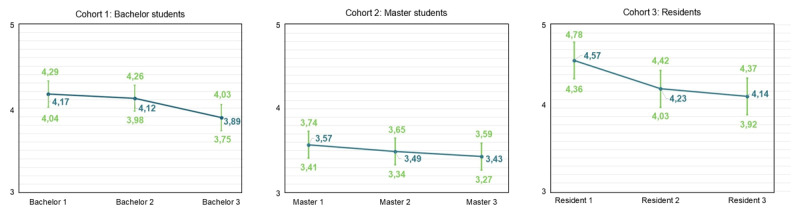
Predicted means pattern for each cohort for engagement (full dataset).

For engagement, results indicate a significant decrease in bachelor year 3 (β = -0.276, SE = 0.077, p < .01), resident year 2 (β = –0.340, SE = 0.093, p < .001) and resident year 3 (β = –0.423, SE = 0.106, p < .001) (Supplementary Table S.14). Predicted means are illustrated in Figure 5. The t-tests with Benjamini-Hochberg correction showed no significant differences between GP residents and other residents (Supplementary Table S.15, Supplementary Figure S.4).

### Main demands, resources and personality traits

**Figure 6:**
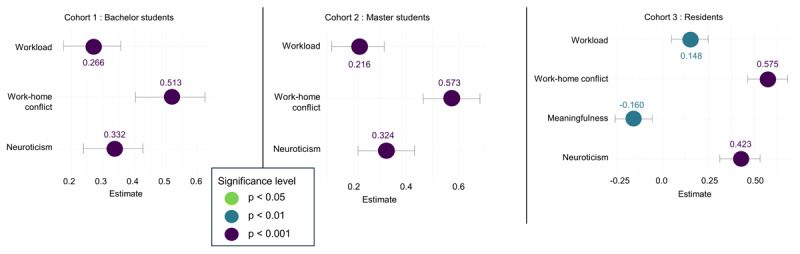
Bee swarm plots with CI of significant determinants of emotional exhaustion per cohort.

Additional LMMs were used to examine which demands (Hypothesis 2a) and resources (Hypothesis 2b) were most strongly associated with emotional exhaustion, cynicism, professional efficacy and engagement within each cohort. For emotional exhaustion, workload, work-home conflict and meaningfulness were significant determinants for all three cohorts, and for cohort 3 meaningfulness was a fourth significant determinant (Supplementary Table S.16). These results are visualized in Figure 6, which presents bee swarm plots with corresponding confidence intervals. Cross-lagged analyses (Supplementary Table S.17) showed that for cohort 1 and cohort 3, neuroticism and work-home conflict remained significant, while for cohort 2, only neuroticism remained significant.

**Figure 7:**
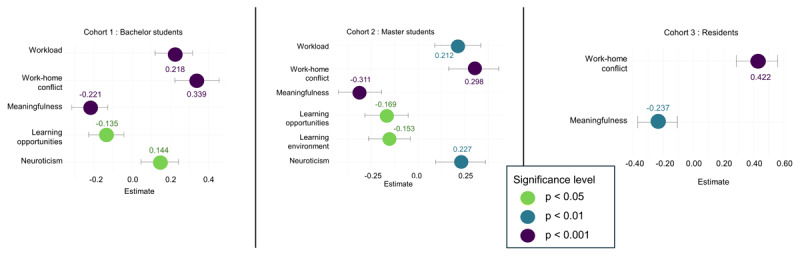
Bee swarm plots with CI of significant determinants of cynicism per cohort.

For cynicism, among cohorts 1 and 2, workload, work-home conflict, meaningfulness, learning opportunities and neuroticism were identified as significant determinants; in cohort 2, the learning environment also remained a significant determinant. For cohort 3, work-home conflict and meaningfulness remained the only significant determinants (Supplementary Table S.18). Figure 7 displays bee swarm plots illustrating these associations along with their corresponding confidence intervals. Cross-lagged analyses (Supplementary Table S.19) indicated that perfectionism was a significant predictor in cohort 1, whereas meaningfulness and neuroticism were significant in cohort 2; in cohort 3, only work–home conflict remained a significant predictor over time.

**Figure 8:**
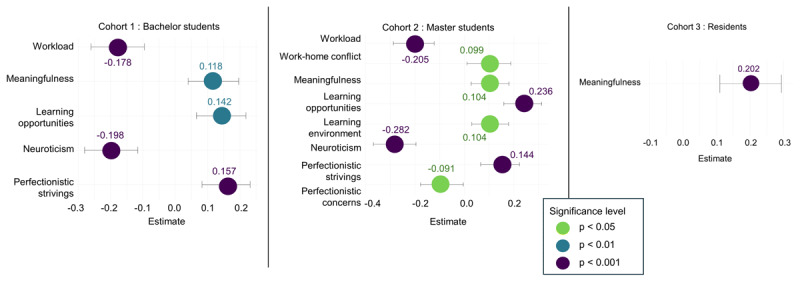
Bee swarm plots with CI of determinants of professional efficacy per cohort.

For professional efficacy, among cohorts 1 and 2, workload, meaningfulness, learning opportunities, neuroticism and perfectionistic strivings were significant determinants. In cohort 2, work-home conflict, learning environment and perfectionistic concerns additionally emerged as significant, whereas in cohort 3, only meaningfulness remained significant (Supplementary Table S.20). An unexpected positive association with work-home conflict prompted the inclusion of an interaction term between workload and work–home conflict, given their high intercorrelation; this interaction was also significant (β = 0.150, SE = 0.067, *p* < .05). Figure 8 presents bee swarm plots depicting these associations with confidence intervals. Cross-lagged analyses (Supplementary Table S.21) identified no significant predictors for cohorts 1 and 3. In cohort 2, however, workload, learning opportunities and neuroticism were positively associated with changes in professional efficacy.

**Figure 9:**
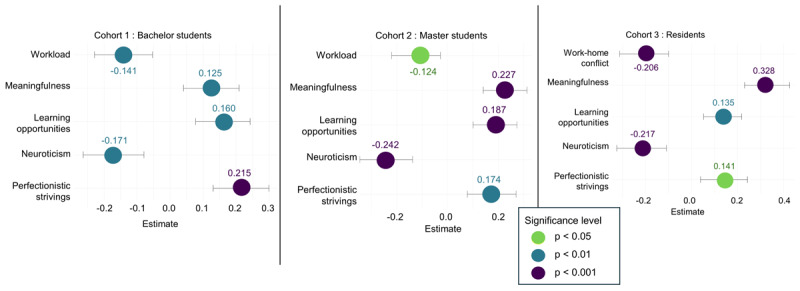
Bee swarm plots with CI of determinants of engagement per cohort.

For engagement, in cohorts 1 and 2, workload, meaningfulness, learning opportunities, neuroticism, and perfectionistic strivings were significant determinants (Supplementary Table S.22). In cohort 3, work-home conflict replaced workload as a significant demand. Figure 9 displays these results in bee swarm plots. Cross-lagged analyses (Supplementary Table S.23) indicated that perfectionistic strivings significantly predicted engagement in cohort 1, learning opportunities in cohort 2, and no significant predictors were identified in cohort 3.

## Discussion

The results indicate a pattern of increasing burnout complaints and declining engagement within medical education learning phases. In general, emotional exhaustion and cynicism increased significantly but modestly within bachelor, master, and resident years, while engagement showed a declining trend, most notably during residency years. Small significant differences in cynicism and professional efficacy were observed between GP residents and other residents, indicating potential specialty-specific patterns. In the preclinical phases, workload and work-home conflict were the primary demands, whereas work-home conflict dominated during residency. Meaningfulness and learning opportunities were the most important resources for students, while meaningfulness was the key resource for residents. Within all cohorts, neuroticism consistently affected both burnout and engagement.

### Modest Increases in Burnout Complaints and Decreases in Engagement

Hypothesis 1a was supported, with modest but significant increases in burnout complaints and decreases in engagement observed within medical education learning phases. These findings align with former studies indicating a progressive rise in burnout complaints accompanied by a declining trend in engagement during medical education [13,14,17,18,19,61,62]. Interestingly, bachelor students showed deterioration mainly in the third year, reflected by increases in emotional exhaustion and cynicism, suggesting either an accumulation of chronic stress in the first two years or a substantial escalation of stress during the third year. Among master students, emotional exhaustion remained stable, whereas cynicism increased and professional efficacy decreased; while among residents, emotional exhaustion rose again and engagement declined. These phase-specific patterns underscore the nuanced and complex trajectories of burnout complaints and engagement in medical education, demonstrating that burnout complaints do not necessarily uniformly decrease or increase over time [63].

### GP Residents Versus Other Residents

Furthermore, Hypothesis 1b was supported, with GP residents reporting significantly lower levels of cynicism and higher professional efficacy compared to other residents. These differences are in line with meta-analytic evidence indicating that family medicine is among the specialties with lower burnout prevalence rates, in contrast to hospital specialties such as radiology and surgery [20]. A potential explanation may lie in differences in work context: GP residents often manage relatively stable patients with chronic conditions in community settings, enabling sustained patient relationships that may protect against burnout [64]. In contrast, hospital-based residents more frequently care for acutely ill and unstable patients with complex complications and face greater exposure to litigation risk, factors that may contribute to higher burnout levels [65]. Additionally, GP training programs in Belgium incorporate designated supervisors, regular mentoring sessions, and allocate one day per week for study, factors that may contribute to enhanced well-being among GP residents [66,67].

### Workload and Work-Home Conflict as Key Demands

Hypothesis 2a was partially supported. As hypothesized, workload was identified as a key demand during preclinical phases, where it was significantly associated with emotional exhaustion, cynicism, and engagement. In the clinical phase, workload remained relevant for emotional exhaustion. However, work–home conflict was a more influential demand for emotional exhaustion and cynicism within all learning phases compared to workload, exceeding our expectation that it would be primarily relevant during residency years. In addition, and contrary to our expectation, cognitive demands were not significant in either the preclinical or residency phases, with workload and work-home conflict proving more salient. Cross-lagged analyses further confirmed the predictive role of work–home conflict for subsequent increases in emotional exhaustion and cynicism, suggesting a potentially causal influence, which is consistent with prior research [68,69,70,71,72,73,74].

In order to understand the importance of work-home conflict within all learning phases, it may be important to situate it within broader societal transformations, including evolving gender roles, generational trends, and the erosion of work-life boundaries, amplified in part by technological advancements [75,76,77,78,79]. As both women and men now increasingly engage in both earning and caregiving roles, the traditional separation between gender roles becomes less clear [77,80]. Further, the generational composition of our samples, largely Generation Z, should also be taken into consideration. While Gen Z is often characterized as prioritizing a healthy work-life balance, working hours flexibility and well-being, studies have shown that life-stage variables, such as parenting status, and societal changes, have a more substantial impact on work-home dynamics than generational effects alone [78,79]. Lastly, digital technologies, despite enabling flexibility, also stimulate constant connectivity, increasing stress and complicating efforts to maintain healthy work–life boundaries [76,81].

### Meaningfulness and Learning Opportunities a Key Resources

Hypothesis 2b received partial support. As anticipated, meaningfulness was the most important resource for residents, whereas for students, meaningfulness and learning opportunities were similarly influential. Specifically, among students, meaningfulness and learning opportunities were strongly associated with cynicism, professional efficacy, and engagement, but not with emotional exhaustion. For residents, meaningfulness was the primary resource linked to all outcomes, with learning opportunities only related to engagement. These findings echo recent evidence demonstrating that meaningful work buffers against burnout and promotes engagement [42,82,83,84,85]. For example, Chênevert et al. (2021) reported that physicians value meaningful work and patient recognition more than general organizational support [85]. They found that time spent on meaningful tasks constitutes a strong protective factor against burnout and also reduces dropout intentions [86,87].

Contrary to expectations, the learning environment was only significantly associated with cynicism and professional efficacy in master students and not with emotional exhaustion or engagement. This finding seems to diverge from existing evidence [36,43,44,45,88,89]. However, this ambiguous pattern may also reflect the complexity of the learning environment in a medical educational context, which comprises multiple distinct components, including academic learning and learning in a clinical context. Each component is characterized by specific factors, such as the educational climate, supervisory presence and style, and alignment of tasks with competency development [36]. These contextual elements were not accounted for in the present analysis. The learning environment is further characterized by less clearly defined teams and fluid relationships among students, residents, and supervisors [90]. Third year master students and residents, for instance, frequently rotate between departments and engage in short-term collaborations with multiple supervisors, which may complicate perceptions about the learning environment. Additionally, uncertainty regarding the appropriate timing or circumstances for approaching supervisors, coupled with concerns about potential negative repercussions related to evaluations may further attenuate the perceived impact of environmental support [90].

### Perfectionism and Neuroticism

Consistent with former studies, neuroticism was associated with higher burnout complaints and lower engagement, a pattern largely confirmed by the cross-lagged analyses, except for engagement. This aligns with prior research presenting neuroticism as an important determinant for poor well-being [24,25,38,61,88]. For instance, Bianchi et al. (2018) reported neuroticism to be more influential than work-related or social factors. Perfectionism showed a more nuanced pattern, being more related to professional efficacy and engagement, particularly through perfectionistic strivings, reflecting a motivation to achieve high standards [91,92]. However, due to insufficient statistical power within the cohorts, we could not examine potential mediating or moderating effects of these personality traits. Therefore, consistent with Robins et al. (2018), caution is necessary when interpreting the direct influence of personality traits, as their mediation analyses suggest a more complex relationship in which personality also shapes demands and resources that subsequently affect burnout and engagement [93].

### Implications for Practice

In a medical education context, strategies to address these key demands and resources, namely work–home conflict, workload, meaningfulness, and learning opportunities, should be tailored to the distinct realities of students and residents. For medical students, in an academic learning environment, interventions may focus on flexible curricular scheduling, protected study time, manageable deadlines, and access to supportive infrastructure such as green spaces and sports facilities, alongside educational designs that emphasize meaningful learning, professional identity formation, and participatory teaching approaches [66,94,95,96]. For residents in clinical settings, structural measures such as flexible and self-scheduling, limits on duty hours supported by adequate staffing, task reallocation through skill-mix teams, and access to sabbaticals can help mitigate work–home conflict and excessive workload, while reducing administrative burden and stimulating interprofessional collaboration and shared decision-making may strengthen meaningfulness in clinical work [66,97,98]. Together, these context-specific strategies acknowledge the differing demands of academic and clinical training environments while addressing core determinants of burnout and engagement across medical education.

### Strengths and Limitations

One of the key strengths of this study is the repeated measures design, which enables the examination of the evolution of burnout complaints and engagement within learning phases of medical education, namely bachelor years, master years, and resident years. This approach allows for a more comprehensive understanding of how burnout complaints and engagement evolve over time, capturing evolutions within learning phases and fluctuations that cross-sectional studies might miss. Another strength is the study’s focus on a broad range of contributing factors, including demands, resources, and personality traits, which provides a nuanced view of which factors are most important. Additionally, the inclusion of multiple cohorts from various medical faculties strengthens the generalizability of the findings, offering insights that can be applied within diverse educational settings. Nevertheless, we should also note several limitations of our research. First, the response rate was low at 14%, despite our continuous recruitment efforts. This may have limited generalizability and suggest that the findings primarily reflect the views of a self-selected sample rather than the entire population. Participants suggested that high workload, time constraints, the sensitive nature of the topic, and survey fatigue may have contributed to limited participation, introducing the potential for participation bias. Sensitivity analyses comparing participants who completed a single survey with those completing multiple surveys using t-tests revealed no significant differences, indicating that attrition did not substantially bias the findings. In addition, our sample did not significantly differ from the population with regard to gender composition. Nevertheless, future studies could mitigate this limitation by integrating survey assessments into the medical curriculum, which would both normalize participation and reduce the risk of selection bias by reaching the full population. Further, the incorporated validated questionnaires (i.e., MBI, COPSOQ) relied on self-report which is prone to social desirability bias, as participants might under- or overreport their burnout complaints and engagement levels. Nonetheless, the use of an anonymous online survey might mitigate this bias to some extent. In addition, it is important to note that our analyses do not diagnose clinical burnout, therefore, our discussions revolve around burnout complaints rather than burnout as defined by clinical criteria. Next, while the design provides valuable insights, the three-year study period may not fully capture the long-term effects of burnout. Continuation of the survey would be recommended for long-term results. Finally, cohort bias should be considered, as differences observed between bachelor, master, and residency cohorts may not solely reflect educational influences but could also be attributed to cohort-specific characteristics.

## Conclusion

This study provides further insights into the dynamics of burnout complaints and engagement among medical students and residents within different learning phases of medical education. The findings indicate a trend of modestly increasing burnout complaints, and decreasing engagement within each learning phase (i.e. bachelor students, master students, and residents). GP residents exhibited a pattern of lower levels of burnout complaints, along with higher engagement, compared to other residents. Workload and work-home conflict were key demands within all cohorts, while meaningfulness was the strongest protective factor, followed by learning opportunities. The influence of personality traits, namely neuroticism and perfectionism, highlights the need for a comprehensive understanding of individual factors that also contribute to burnout complaints and engagement. Efforts should prioritize reducing perceived workload and work–home conflict, while enhancing meaningfulness and learning opportunities.

## Data Accessibility Statement

The datasets used and/or analyzed during the current study are available from the corresponding author on reasonable request.

## Additional File

The additional file for this article can be found as follows:

10.5334/pme.2013.s1 Supplementary File. Supplementary Tables S.1 to S.23 and Figures S.1 to S.4.

## Abbreviations

BFI-XS: Big Five Inventory-Extra Short

COPSOQ: Copenhagen Psychosocial Questionnaire

DF: Degrees of Freedom

FMPS: Frost Multidimensional Perfectionism Scale

FIML: Full Information Maximum Likelihood

GP: General Practice

JD-R: Job Demands – Resources

LMM: Linear Mixed-effects Models

M: Mean

MBI-SS: Maslach Burnout Inventory – Student Survey

MCAR: Missing Completely At Random

SD: Standard Deviation

UBOS-C: Utrecht Burnout Scale – Contact-based professions

UWES: Utrecht Work Engagement Scale

UWES-S: Utrecht Work Engagement Scale – Students
