# Supplementary material for: Burnout and Engagement Within Medical Education: A Repeated Measures Study on Their Evolution and Main Determinants
Source: Perspect Med Educ. 2026 Feb 16;15(1):134–49. doi: 10.5334/pme.2013 (PMC12922681; doi:10.5334/pme.2013)

@spl msr_l b cl e_eck cl r u gfgl k cbga_j cbsa_rgml 8_ pcnc_rcb k c_qspcq qrsbw

ml rf cgpct mjsrgml _l b k _gl bcrcpk gl _l rq,

Supplementary Tables

Table S.1 T-test results MCAR for emotional exhaustion, cynicism, professional efficacy and engagement

**Emotional exhaustion**	**Mean (SD)**	**CI (95%)**	**t-ratio (df)**	**p-value**
**Bachelor and master students (cohort 1 and 2)**				
Present at T1 only	2.71 (1.23)			
Present T1 and T2	2.71 (1.21)	-0.27 to 0.27	0.01 (309)	0.996
Present T2 only	3.10 (1.37)			
Present T2 and T3	2.88 (1.23)	-0.50 to 0.06	-1.54 (329)	0.124
**Residents (cohort 3)**				
Present at T1 only	2.50 (1.21)			
Present T1 and T2	2.72 (1.43)	-0.26 to 0.70	0.90 (116)	0.369
Present T2 only	2.99 (1.35)			
Present T2 and T3	3.01 (1.36)	-0.48 to 0.52	0.08 (118)	0.934

**Cynicism**	**Mean (SD)**	**CI (95%)**	**t-ratio (df)**	**p-value**
**Bachelor and master students (cohort 1 and 2)**				
Present at T1 only	1.01 (1.09)			
Present T1 and T2	1.21 (1.27)	-0.06 to 0.47	1.52 (309)	0.130
Present T2 only	1.51 (1.32)			
Present T2 and T3	1.59 (1.41)	-0.37 to 0.21	-0.55 (329)	0.583
**Residents (cohort 3)**				
Present at T1 only	1.88 (1.18)			
Present T1 and T2	1.72 (1.29)	-0.62 to 0.29	-0.73 (116)	0.471
Present T2 only	2.09 (1.26)			
Present T2 and T3	1.80 (1.08)	-0.73 to 0.15	-1.30 (118)	0.196

**Professional efficacy**	**Mean (SD)**	**CI (95%)**	**t-ratio (df)**	**p-value**
**Bachelor and master students (cohort 1 and 2)**				
Present at T1 only	3.72 (0.84)			
Present T1 and T2	3.76 (0.91)	-0.16 to 0.23	0.29 (309)	0.768
Present T2 only	3.29 (0.99)			
Present T2 and T3	3.45 (0.87)	-0.05 to 0.36	1.51 (329)	0.131
**Residents (cohort 3)**				
Present at T1 only	4.87 (0.86)			
Present T1 and T2	5.11 (0.76)	-0.07 to 0.53	1.51 (116)	0.133
Present T2 only	4.86 (0.87)			
Present T2 and T3	4.88 (0.81)	-0.28 to 0.34	0.18 (118)	0.856

**Engagement**	**Mean (SD)**	**CI (95%)**	**t-ratio (df)**	**p-value**
**Bachelor and master students (cohort 1 and 2)**				
Present at T1 only	4.09 (0.95)			
Present T1 and T2	4.03 (1.12)	-0.29 to 0.17	-0.49 (309)	0.624
Present T2 only	3.67 (1.18)			
Present T2 and T3	3.90 (1.08)	-0.02 to 0.47	1.81 (329)	0.071
**Residents (cohort 3)**				
Present at T1 only	4.57 (0.97)			
Present T1 and T2	4.67 (1.04)	-0.27 to 0.47	0.55 (116)	0.585
Present T2 only	4.22 (0.98)			
Present T2 and T3	4.22 (0.99)	-0.36 to 0.37	0.02 (118)	0.987

Table S.2 Background information of the participants (full dataset)

Cohort	Study Year	Time	Age		Gender (Female)		Children (No)		Nationality (Belgian)		Living Situation						Total
											Alone		With partner or friends		With parents		
			Mean	SD	N	%	N	%	N	%	N	%	N	%	N	%	N
**Cohort 1**	**Bachelor 1**	**T0**	18.63	2.40	142	72.08	195	98.98	192	97.46	6	3.05	11	5.58	180	91.37	197
	**Bachelor 2**	**T1**	19.65	2.49	127	79.87	158	99.37	151	94.94	6	3.77	15	9.43	138	86.79	159
	**Bachelor 3**	**T2**	20.61	2.20	105	68.18	153	99.35	148	96.10	8	5.19	13	8.44	133	86.36	154

**Cohort 2**	**Master 1**	**T0**	21.77	2.01	83	72.81	112	98.25	111	97.37	5	4.39	21	18.42	88	77.19	114
	**Master 2**	**T1**	22.90	2.67	121	70.35	167	97.09	165	95.93	6	3.49	32	18.60	134	77.91	172
	**Master 3**	**T2**	23.96	2.35	103	75.74	134	98.53	133	97.79	3	2.21	30	22.06	103	75.74	136
**Cohort 3**	**Resident 1**	**T0**	25.58	3.04	89	75.42	113	95.76	112	94.92	14	11.86	76	64.41	28	35.82	118
	**Resident 2**	**T1**	26.51	3.84	82	67.21	115	94.26	121	99.18	17	13.93	81	66.39	24	19.67	122
	**Resident 3**	**T2**	27.39	3.93	65	76.47	80	94.12	84	98.82	15	17.65	61	71.76	9	10.59	85
**Total**			**22.73**	**4.05**	**917**	**72.15**	**1227**	**96.87**	**1217**	**96.82**	**80**	**8.07**	**340**	**36.08**	**837**	**60.04**	**1257**

Table S.3 Background information of the participants (complete dataset)

**Cohort**	**Study Year**	**Time**	**Age**		**Gender (Female)**		**Children (No)**		**Nationality (Belgian)**		**Living Situation**						**Total**
											**Alone**		**With partner or friends**		**With parents**		
			**Mean**	**SD**	**N**	**%**	**N**	**%**	**N**	**%**	**N**	**%**	**N**	**%**	**N**	**%**	**N**
**Cohort 1**	**Bachelor 1**	**T0**	18.98	3.23	47	77.05	60	98.36	60	98.36	2	3.28	4	6.56	55	90.16	61
	**Bachelor 2**	**T1**	19.95	3.24	47	77.05	60	98.36	60	98.36	3	4.92	9	14.75	49	80.33	61
	**Bachelor 3**	**T2**	21.05	3.21	46	75.41	60	98.36	60	98.36	2	3.28	7	11.48	52	85.25	61
**Cohort 2**	**Master 1**	**T0**	21.60	2.06	36	75.00	47	97.92	47	97.92	0	0.00	8	16.67	40	83.33	48
	**Master 2**	**T1**	22.54	2.09	36	75.00	47	97.92	47	97.92	1	2.08	7	14.58	40	83.33	48
	**Master 3**	**T2**	23.60	2.08	36	75.00	47	97.92	47	97.92	1	2.08	6	12.50	41	85.42	48
**Cohort 3**	**Resident 1**	**T0**	25.70	3.21	24	72.73	32	96.97	33	100	2	6.06	22	66.67	9	27.27	33
	**Resident 2**	**T1**	26.52	3.30	24	72.73	31	93.94	33	100	3	9.09	24	72.73	6	18.18	33
	**Resident 3**	**T2**	27.55	3.30	24	72.73	30	90.91	33	100	5	15.15	23	69.70	5	15.15	33
**Total**			**22.41**	**3.94**	**320**	**75.12**	**414**	**97.18**	**420**	**98.59**	**19**	**4.46**	**110**	**25.82**	**297**	**69.72**	**426**

Table S.4 Background information of Cohort 3, divided into GP residents and other residents for the full and complete datasets

Full dataset

**Cohort 3: Residents**	**Time**	**Age**		**Gender (Female)**		**Children (No)**		**Nationality (Belgian)**		**Living Situation**						**Total**
										**Alone**		**With partner or friends**		**With parents**		
		**Mean**	**SD**	**N**	**%**	**N**	**%**	**N**	**%**	**N**	**%**	**N**	**%**	**N**	**%**	**N**
**GP resident 1**	**T0**	25.9	3.33	36	75	45	93.75	47	97.92	3	6.25	28	58.33	17	35.42	48
**GP resident 2**	**T1**	26.6	3.91	44	75.86	54	93.10	58	100	6	10.34	35	60.34	17	29.31	58
**GP resident 3**	**T2**	27.5	4.54	38	84.44	43	95.56	44	97.78	7	15.56	31	68.89	7	15.56	45
**Other resident 1**	**T0**	25.4	2.84	53	75.71	68	97.14	65	92.86	11	15.71	48	68.57	11	15.71	70
**Other resident 2**	**T1**	26.4	3.81	38	59.38	61	95.31	63	98.44	11	17.19	46	71.88	7	10.94	64
**Other resident 3**	**T2**	27.2	3.14	27	67.50	37	92.50	40	100	8	20	30	75	2	5	40
**Total**		**26.4**	**3.66**	**236**	**72.62**	**308**	**94.77**	**317**	**97.54**	**46**	**14.15**	**218**	**67.08**	**61**	**18.77**	**325**

Complete dataset

**Cohort 3: Residents**	**Time**	**Age**		**Gender (Female)**		**Children (No)**		**Nationality (Belgian)**		**Living Situation**						**Total**
										**Alone**		**With partner or friends**		**With parents**		
		**Mean**	**SD**	**N**	**%**	**N**	**%**	**N**	**%**	**N**	**%**	**N**	**%**	**N**	**%**	**N**
**GP resident 1**	**T0**	25.31	1.40	13	81.25	16	100	16	100	0	0	9	56.25	7	43.75	16
**GP resident 2**	**T1**	26.06	1.39	15	83.33	18	100	18	100	1	5.56	11	61.11	6	33.33	18
**GP resident 3**	**T2**	27.06	1.51	15	83.33	18	100	18	100	2	11.11	11	61.11	5	27.78	18
**Other resident 1**	**T0**	26.06	4.29	11	64.71	16	94.12	17	100	2	11.11	13	76.47	2	11.76	17
**Other resident 2**	**T1**	27.07	4.68	9	60.0	13	86.67	15	100	2	13.33	13	86.67	0	0	15
**Other resident 3**	**T2**	28.13	4.63	9	60.0	12	80	15	100	3	20	12	80	0	0	15
**Total**		**26.61**	**1.02**	**72**	**72.73**	**93**	**93.93**	**99**	**100**	**10**	**10.10**	**69**	**69.68**	**20**	**20.20**	**99**

Table S.5 Descriptives on emotional exhaustion, cynicism, professional efficacy, and engagement (full dataset)

**Cohort**	**Study year**	**Time**	**N**	**%Missing**	**Mean**	**SD**	**Min**	**Max**	**Skewness**	**Kurtosis**	**Cronbach's Alpha**
					**Emotional Exhaustion**						
**Cohort 1**	**Bachelor 1**	T0	197	0.00	2.57	1.20	0.20	5.60	0.32	-0.69	0.87
	**Bachelor 2**	T1	159	0.00	2.83	1.25	0.40	6.00	0.46	-0.30	0.89
	**Bachelor 3**	T2	154	0.00	2.91	1.35	0.00	6.00	0.34	-0.45	0.92
**Cohort 2**	**Master 1**	T0	114	0.00	2.95	1.23	0.40	5.80	0.21	-0.84	0.89
	**Master 2**	T1	172	0.00	3.16	1.34	0.00	6.00	0.01	-0.65	0.91
	**Master 3**	T2	136	0.00	3.03	1.19	0.40	5.60	0.19	-0.75	0.87
**Cohort 3**	**Resident 1**	T0	118	0.00	2.60	1.31	0.38	6.00	0.51	-0.54	0.91
	**Resident 2**	T1	120	1.64	3.00	1.35	0.62	5.88	0.27	-0.97	0.92

	**Resident 3**	T2	83	2.35	3.22	1.28	0.62	5.88	0.18	-0.66	0.91
					**Cynicism**						
**Cohort 1**	**Bachelor 1**	T0	197	0.00	0.89	1.05	0.00	5.25	1.57	2.90	0.79
	**Bachelor 2**	T1	159	0.00	1.09	1.13	0.00	5.25	1.33	1.66	0.81
	**Bachelor 3**	T2	154	0.00	1.36	1.32	0.00	6.00	1.13	0.73	0.86
**Cohort 2**	**Master 1**	T0	114	0.00	1.50	1.32	0.00	5.25	0.87	-0.03	0.85
	**Master 2**	T1	172	0.00	1.99	1.43	0.00	5.75	0.50	-0.70	0.84
	**Master 3**	T2	136	0.00	1.96	1.37	0.00	5.75	0.81	0.16	0.86
**Cohort 3**	**Resident 1**	T0	118	0.00	1.82	1.23	0.00	5.00	0.76	-0.34	0.76
	**Resident 2**	T1	120	1.64	1.97	1.19	0.00	5.20	0.53	-0.44	0.74
	**Resident 3**	T2	83	2.35	2.03	1.23	0.40	5.40	0.67	-0.37	0.78
					**Professional Efficacy**						
**Cohort 1**	**Bachelor 1**	T0	197	0.00	3.79	0.84	1.40	6.00	-0.28	0.01	0.69
	**Bachelor 2**	T1	159	0.00	3.53	0.97	1.00	6.00	-0.40	-0.01	0.80
	**Bachelor 3**	T2	154	0.00	3.39	0.86	1.17	5.67	-0.33	-0.35	0.73
**Cohort 2**	**Master 1**	T0	114	0.00	3.66	0.93	1.80	5.80	-0.03	-0.56	0.79
	**Master 2**	T1	172	0.00	3.21	0.89	1.17	5.50	0.09	-0.44	0.75
	**Master 3**	T2	136	0.00	3.26	0.87	1.17	5.83	0.24	-0.21	0.76
**Cohort 3**	**Resident 1**	T0	118	0.00	4.98	0.82	1.71	6.00	-1.23	1.64	0.83
	**Resident 2**	T1	120	1.64	4.87	0.85	2.43	6.00	-0.78	0.11	0.82
	**Resident 3**	T2	83	2.35	5.04	0.74	2.14	6.00	-1.16	1.86	0.83
					**Engagement**						
**Cohort 1**	**Bachelor 1**	T0	197	0.00	4.28	0.96	1.11	6.00	-0.79	0.46	0.89
	**Bachelor 2**	T1	159	0.00	4.13	1.05	0.67	6.00	-0.76	0.55	0.89
	**Bachelor 3**	T2	154	0.00	3.93	1.03	0.78	6.00	-0.39	-0.25	0.88
**Cohort 2**	**Master 1**	T0	114	0.00	3.68	1.07	1.11	5.67	-0.13	-0.60	0.91
	**Master 2**	T1	172	0.00	3.46	1.12	0.56	5.78	-0.16	-0.63	0.89
	**Master 3**	T2	136	0.00	3.43	1.09	1.22	5.89	0.07	-0.76	0.90
**Cohort 3**	**Resident 1**	T0	118	0.00	4.61	1.00	1.22	6.00	-1.43	2.40	0.92
	**Resident 2**	T1	120	1.64	4.22	0.98	1.11	5.89	-0.66	-0.01	0.89
	**Resident 3**	**T2**	83	2.35	4.06	1.05	0.33	5.89	-0.84	0.97	0.91

Note. Skewness > 2 and kurtosis > 7 indicate substantial non-normality (Kim, 2013).

Table S.6 Descriptives on emotional exhaustion, cynicism, professional efficacy, and engagement (complete dataset)

Cohort	Study year	Time	N	%Missing	Mean	SD	Min	Max	Skewness	Kurtosis	Cronbach's Alpha
					**Emotional Exhaustion**						
**Cohort 1**	**Bachelor 1**	T0	61	0.00	2.61	1.22	0.40	5.20	0.28	-0.79	0.88
	**Bachelor 2**	T1	61	0.00	2.74	1.14	1.00	5.80	0.69	-0.07	0.86
	**Bachelor 3**	T2	61	0.00	2.91	1.31	0.00	6.00	0.41	-0.20	0.93
**Cohort 2**	**Master 1**	T0	48	0.00	2.79	1.17	0.60	5.20	0.34	-1.01	0.89
	**Master 2**	T1	48	0.00	2.82	1.27	0.00	5.80	0.12	-0.51	0.92
	**Master 3**	T2	48	0.00	2.74	1.05	0.40	5.60	0.34	0.01	0.86
**Cohort 3**	**Resident 1**	T0	33	0.00	3.00	1.45	0.50	5.62	-0.10	-1.35	0.92
	**Resident 2**	T1	33	0.00	3.14	1.40	0.75	5.88	0.08	-1.15	0.93
	**Resident 3**	T2	33	0.00	2.97	1.22	0.62	5.38	0.07	-0.76	0.9
					**Cynicism**						
**Cohort 1**	**Bachelor 1**	T0	61	0.00	0.98	1.22	0.00	5.25	1.56	2.40	0.82
	**Bachelor 2**	T1	61	0.00	1.05	1.08	0.00	4.25	1.05	0.22	0.77
	**Bachelor 3**	T2	61	0.00	1.37	1.35	0.00	5.00	1.03	0.07	0.87
**Cohort 2**	**Master 1**	T0	48	0.00	1.47	1.26	0.00	5.25	0.95	0.46	0.82
	**Master 2**	T1	48	0.00	1.79	1.30	0.00	5.00	0.89	-0.07	0.87
	**Master 3**	T2	48	0.00	1.65	1.19	0.00	5.75	1.20	1.94	0.85
**Cohort 3**	**Resident 1**	T0	33	0.00	1.82	1.35	0.20	5.00	0.86	-0.59	0.86
	**Resident 2**	T1	33	0.00	1.91	1.13	0.00	4.20	0.40	-0.86	0.75
	**Resident 3**	T2	33	0.00	1.69	1.14	0.40	5.40	1.23	1.42	0.81
					**Professional Efficacy**						
**Cohort 1**	**Bachelor 1**	T0	61	0.00	3.98	0.87	2.00	5.60	-0.46	-0.38	0.75
	**Bachelor 2**	T1	61	0.00	3.73	0.91	1.67	5.67	-0.42	-0.47	0.81
	**Bachelor 3**	T2	61	0.00	3.48	0.87	1.67	5.67	-0.38	-0.32	0.77
**Cohort 2**	**Master 1**	T0	48	0.00	3.59	0.87	1.80	5.20	-0.28	-0.58	0.82
	**Master 2**	T1	48	0.00	3.27	0.83	1.50	5.33	0.16	-0.55	0.74
	**Master 3**	T2	48	0.00	3.37	0.82	1.83	5.17	0.28	-0.67	0.74
**Cohort 3**	**Resident 1**	T0	33	0.00	5.11	0.76	2.71	6.00	-1.48	2.13	0.82
	**Resident 2**	T1	33	0.00	5.02	0.80	3.14	6.00	-0.67	-0.62	0.8
	**Resident 3**	T2	33	0.00	5.32	0.51	4.43	6.00	-0.48	-1.16	0.77

	Engagement										
**Cohort 1**	**Bachelor 1**	T0	61	0.00	4.45	1.06	1.11	6.00	-0.91	0.54	0.91
	**Bachelor 2**	T1	61	0.00	4.39	0.88	2.22	5.89	-0.36	-0.86	0.87
	**Bachelor 3**	T2	61	0.00	4.14	0.98	1.78	6.00	-0.47	-0.39	0.88
**Cohort 2**	**Master 1**	T0	48	0.00	3.57	1.12	1.11	5.67	-0.15	-0.62	0.92
	**Master 2**	T1	48	0.00	3.60	1.03	1.56	5.44	0.00	-0.93	0.89
	**Master 3**	T2	48	0.00	3.63	1.04	1.33	5.67	-0.13	-0.76	0.9
**Cohort 3**	**Resident 1**	T0	33	0.00	4.61	1.07	1.33	6.00	-1.40	2.15	0.93
	**Resident 2**	T1	33	0.00	4.39	0.96	2.22	5.89	-0.78	-0.15	0.87
	**Resident 3**	**T2**	33	0.00	4.31	0.91	2.00	5.89	-0.47	-0.16	0.85

Note. Skewness > 2 and kurtosis > 7 indicate substantial non-normality (Kim, 2013).

R_` jc Q,5 Amppcj_rgml r_` jcq ncpamf mpr &dsjj b_r_qcr'

Amf mpr / 8@_af cjmpqrsbcl rq

Variable	1	2	3	4	5	6	7	8	9	10	11	12	13	14	15	16
**1. Emotional exhaustion**		.63***	-.31***	-.37***	.01	.01	.11*	.21***	.53***	.63***	-.20***	-.21***	-.18***	.46***	.09*	.31***
**2. Cynicism**			-.37***	-.44***	-.03	-.02	.16***	.07	.47***	.50***	-.40***	-.29***	-.23***	.30***	-.04	.21***
**3. Professional efficacy**				.65***	.01	-.19***	-.19***	.04	-.33***	-.15***	.34***	.32***	.20***	-.31***	.16***	-.11*
**4. Engagement**					.05	-.12**	-.14**	.12**	-.27***	-.14**	.34***	.34***	.11*	-.22***	.26***	.04
**5. Age**						.02	.33***	.12**	.18***	.21***	.00	.04	-.18***	-.06	-.06	.03
**6. Gender**							-.02	.01	.10*	-.03	.01	.06	-.04	.29***	.04	.08
**7. Study year**								.14**	.04	.13**	-.02	.00	-.29***	.05	-.02	.06
**8. Cognitive demands**									.18***	.35***	.19***	.25***	-.23***	.12**	.14**	.22***
**9. Workload**										.54***	-.23***	-.15***	-.21***	.26***	-.04	.15***
**10. Work-home conflict**											-.17***	-.11*	-.26***	.31***	.19***	.27***
**11. Meaningfulness**												.45***	.13**	-.15***	.10*	.01
**12. Learning opportunities**													.04	-.08	.09*	.00
**13. Learning environment**														-.19***	-.04	-.19***
**14. Neuroticism**															.16***	.40***
**15. Perfectionistic strivings**																.41***
**16. Perfectionistic concerns**																

Note: Significance levels at *p<0.05; **p<0.01,***p<0.001

Amf mpr 08K_qrcpqrsbcl rq

Variable	1	2	3	4	5	6	7	8	9	10	11	12	13	14	15	16
**1. Emotional exhaustion**		,46(((	-,25(((	-,22(((	,. 3	,/ 5(((	,. 0	,/ 5(((	,30(((	,41(((	-,04(((	-,0/ (((	-,0. (((	,33(((	,/ 1((	,15(((
**2. Cynicism**			-,31(((	-,31(((	,. 7	,. 1	,/ 0(	,. 5	,2. (((	,20(((	-,23(((	-,14(((	-,01(((	,14(((	,. 2	,01(((
**3. Professional efficacy**				,45(((	-,. 3	-,0/ (((	-,/ 4((	,. 0	-,16(((	-,0. (((	,17(((	,2/ (((	,/ 5(((	-,2. (((	,/ 1((	-,0/ (((
**4. Engagement**					-,. 5	-,/ 2((	-,. 7	,. 7	-,07(((	-,/ 6(((	,22(((	,2. (((	,/ 1((	-,1/ (((	,/ 7(((	-,. 5
**5. Age**						,. 1	,11(((	,. 4	,. 4	,/ 6(((	-,. 7	,. 2	-,. 1	,. .	,. .	-,. /
**6. Gender**							,. .	,. /	,/ 1((	,. 4	-,. 0	,. 6	-,. 2	,04(((	-,. 4	,. 7
**7. Study year**								,/ 1((	-,/ / (	,/ / (	-,/ 3((	-,. 0	-,. 6	-,/ 0(	-,. 4	,. 0
**8. Cognitive demands**									,. 6	,11(((	,0. (((	,/ 7(((	-,/ 6(((	,/ 1((	,/ 6(((	,0/
**9. Workload**										,22(((	-,0/	-,/ 3	-,/ 6	,14(((	,. 2	,0/ (((
**10. Work-home conflict**											-,/ 1((	-,. 6	-,04(((	,20(((	,06(((	,11(((
**11. Meaningfulness**												,31(((	,. 4	-,. 7	,/ 6(((	-,. 3
**12. Learning opportunities**													,. 4	-,. 6	,/ . (	-,. 5
**13. Learning environment**														-,. 7	-,/ / (	-,/ 1((
**14. Neuroticism**															,02(((	,26(((
**15. Perfectionistic strivings**																,24(((
**16. Perfectionistic concerns**																

Note: Significance levels at *p<0.05; **p<0.01,***p<0.001

Amf mpr 18Pcqgbcl rq

Variable	1	2	3	4	5	6	7	8	9	10	11	12	13	14	15	16
**1. Emotional exhaustion**		,40(((	-,10(((	-,37(((	-,. /	,/ 6((	,/ 7(((	,00(((	,3. (((	,51(((	-,2. (((	-,1. (((	-,1/ (((	,34(((	,02(((	,13(((
**2. Cynicism**			-,05(((	-,20(((	-,. 6	-,. 3	,. 5	,. 6	,1/ (((	,3. (((	-,13(((	-,04(((	-,/ 7(((	,00(((	,. 7	,0/ (((
**3. Professional efficacy**				,32(((	-,01(((	-,. 5	,. 0	-,. 0	-,/ 6(((	-,07(((	,16(((	,06(((	,/ 0(	-,01(((	-,/ 2(	-,0/ (((
**4. Engagement**					-,. 3	-,/ 1(	-,00(((	-,. /	-,05(((	-,22(((	,32(((	,21(((	,00(((	-,2. (((	,. 0	-,/ 6((
**5. Age**						-,00(((	,/ 7(((	,. 0	,. 1	-,. /	-,. 1	-,/ 4((	,. /	-,. 1	,. 7	-,. 5
**6. Gender**							-,. /	,. /	,. 2	,/ /	-,. 5	-,. 0	-,. 1	,13(((	,/ 6((	,10(((

	1	2	3	4	5	6	7	8	9	10	11	12	13	14	15	16
**7. Study year**								,. 3	,/ 4((	,/ 2(	-,. 6	-,/ 1(	-,/ 4((	,. 0	,. .	,. 1
**8. Cognitive demands**									,14(((	,10(((	,. 1	,. 4	-,/ 1(	,/ 4((	,01(((	,/ 6((
**9. Workload**										,4/ (((	-,/ 1(	-,/ 3((	-,12(((	,/ 7(((	,/ 6((	,/ 4((
**10. Work-home conflict**											-,07(((	-,0/ (((	-,10(((	,17(((	,05(((	,06(((
**11. Meaningfulness**												,25(((	,0/ (((	-,01(((	,. 1	-,. 6
**12. Learning opportunities**													,0. (((	-,/ 4((	,. 1	-,. 2
**13. Learning environment**														-,/ 0(	-,. 5	-,. 0
**14. Neuroticism**															,14(((	,25(((
**15. Perfectionistic strivings**																,26(((
**16. Perfectionistic concerns**																

Note: Significance levels at *p<0.05; **p<0.01,***p<0.001

Table S.8 Evolution of emotional exhaustion in three cohorts (full and complete datasets)

Cohort 1	Full dataset			Complete dataset		
	B (SE)	CI (95%)		B (SE)	CI (95%)	
		Lower	Upper		Lower	Upper
(Intercept)	2.401***	2.112	2.690	2.491***	1.925	3.057
	(0.148)			(0.292)		
Bachelor 2	0.128	-0.046	0.303	0.138	-0.110	0.385
	(0.089)			(0.128)		
Bachelor 3	0.267*	0.061	0.473	0.297	-0.006	0.601
	(0.105)			(0.157)		
Female	0.083	-0.205	0.371	-0.255	-0.816	0.306
	(0.147)			(0.290)		
ICC	0.9361			0.8726		
Observations	510			183		
Log Likelihood	-765.564			-250.083		
AIC	1,561.129			530.166		
BIC	1,624.645			578.308		

Cohort 2	Full dataset			Complete dataset		
	B (SE)	CI (95%)		B (SE)	CI (95%)	
		Lower	Upper		Lower	Upper
(Intercept)	2.747***	2.383	3.111	0.029	2.151	3.354
	(0.186)			(0.151)		
Master 2	0.132	-0.079	0.343	-0.050	-0.263	0.321
	(0.108)			(0.179)		
Master 3	0.059	-0.189	0.307	0.218	-0.396	0.296
	(0.127)			(0.308)		
Female	0.355	0.036	0.673	2.752	-0.387	0.823
	(0.163)			(0.311)		
ICC	0.9216			0.8493		
Observations	422			144		
Log Likelihood	-654.283			-200.393		
AIC	1,336.566			428.787		
BIC	1,393.196			470.364		

Cohort 3	Full dataset			Complete dataset		
	B (SE)	CI (95%)		B (SE)	CI (95%)	
		Lower	Upper		Lower	Upper
(Intercept)	2.124***	1.757	2.491	2.987***	2.130	3.843
	(0.188)			(0.445)		
Resident 2	0.345**	0.117	0.574	0.140	-0.177	0.457
	(0.116)			(0.165)		
Resident 3	0.468**	0.204	0.732	-0.034	-0.398	0.329
	(0.135)			(0.189)		
Female	0.558**	0.191	0.924	0.161	-0.742	1.064
	(0.188)			(0.457)		
ICC	0.9726			0.9406		
Observations	321			99		
Log Likelihood	-497.521			-137.635		
AIC	1,023.042			303.269		
BIC	1,075.843			339.601		

Note: B = Beta Coefficient, SE = Standard Error, CI = Confidence Interval, *p<0.05; **p<0.01, ***p<0.001, Benjamini-Hochberg correction for multiple comparisons

Table S.9 T-test results for emotional exhaustion: GP residents versus other residents (full dataset)

T-Test	B (SE)	df	t.ratio	p.value*
Year 1	-0.200 (0.217)	105	-0.920	0.48
Year 2	0.148 (0.211)	105	0.703	0.48
Year 3	0.487 (0.226)	105	2.156	0.10

Note: B = Beta Coefficient, SE = Standard Error, df = Degrees of Freedom, *Benjamini-Hochberg correction for multiple comparisons

Table S.10 Evolution of cynicism in three cohorts (full and complete datasets)

Cohort 1	Full dataset			Complete dataset		
	B (SE)	CI (95%)		B (SE)	CI (95%)	
		Lower	Upper		Lower	Upper
(Intercept)	0.854***	0,604	1,105	1.197***	0,676	1,718
	(0.128)			(0.269)		
Bachelor 2	0.095	-0,084	0,274	0.07	-0,21	0,35
	(0.092)			(0.145)		
Bachelor 3	0.394***	0,182	0,606	0.387	0,072	0,702
	(0.108)			(0.163)		
Female	-0.004	-0,255	0,246	-0.38	-0,889	0,129
	0.128			(0.263)		
ICC	0.9089			0.8486		
Observations	510			183		
Log Likelihood	-741.098			-255.668		
AIC	1512.196			541.335		
BIC	1575.713			589.478		

Cohort 2	Full dataset			Complete dataset		
	B (SE)	CI (95%)		B (SE)	CI (95%)	
		Lower	Upper		Lower	Upper
(Intercept)	1.597***	1,201	1,994	1.422***	0,74	2,105
	(0.203)			(0.352)		
Master 2	0.408***	0,224	0,591	0.323*	0,083	0,563
	(0.094)			(0.124)		
Master 3	0.409**	0,148	0,67	0.182	-0,18	0,543
	(0.133)			(0.186)		
Female	0.00	-0,358	0,358	0.381	-0,32	1,081
	(0.183)			(0.356)		
ICC	0.933			0.8793		
Observations	422			144		
Log Likelihood	-676.796			-201.422		
AIC	1381.592			430.843		
BIC	1438.222			472.42		

Cohort 3	Full dataset			Complete dataset		
	B (SE)	CI (95%)		B (SE)	CI (95%)	
		Lower	Upper		Lower	Upper
(Intercept)	1.939***	1,593	2,286	2.011***	1,263	2,759
	(0.178)			(0.388)		
Resident 2	0.193	-0,047	0,433	0.091	-0,259	0,441
	(0.122)			(0.182)		
Resident 3	0.231	-0,032	0,495	-0.127	-0,457	0,202
	(0.134)			(0.171)		
Female	-0.136	-0,479	0,207	-0.159	-0,948	0,63
	(0.176)			(0.399)		
ICC	0.9341			0.8911		
Observations	321			99		
Log Likelihood	-485.986			-131.242		
AIC	999.972			290.484		
BIC	1052.772			326.816		

Note: B = Beta Coefficient, SE = Standard Error, CI = Confidence Interval, *p<0.05; **p<0.01, ***p<0.001, Benjamini-Hochberg correction for multiple comparisons

Table S.11 T-test results for cynicism: GP residents versus other residents (full dataset)

T-Test	B (SE)	df	t.ratio	p.value*
Year 1	0.307 (0.212)	105	1.447	0.151
Year 2	0.528 (0.199)	105	2.660	0.027
Year 3	0.399 (0.236)	105	1.694	0.140

Note: B = Beta Coefficient, SE = Standard Error, df = Degrees of Freedom, *Benjamini-Hochberg correction for multiple comparisons

Table S.12 Evolution of professional efficacy in three cohorts (full and complete datasets)

Cohort 1	Full dataset			Complete dataset			Cohort 2	Full dataset			Complete dataset			Cohort 3	Full dataset			Complete dataset		
	B (SE)	CI (95%)		B (SE)	CI (95%)			B (SE)	CI (95%)		B (SE)	CI (95%)			B (SE)	CI (95%)		B (SE)	CI (95%)	
		Lower	Upper		Lower	Upper			Lower	Upper		Lower	Upper			Lower	Upper		Lower	Upper
(Intercept)	4.071***	3,878	4,263	4.223***	3,843	4,603	(Intercept)	3.975***	3,714	4,237	4.002***	3,554	4,451	(Intercept)	5.063***	4,832	5,295	4.882***	4,505	5,259
	(0.099)			(0.196)				(0.134)			(0.232)				(0.119)			(0.196)		
Bachelor 2	-0.195*	-0,336	-0,054	-0.251*	-0,427	-0,076	Master 2	-0.388***	-0,53	-0,25	-0.313*	-0,53	-0,099	Resident 2	-0.123	-0,275	0,029	-0.091	-0,328	0,147
	(0.072)			(0.091)				(0.072)			(0.111)				(0.077)			(0.123)		
Bachelor 3	-0.391**	-0,541	-0,24	-0.504***	-0,742	-0,266	Master 3	-0.343***	-0,517	-0,17	-0.219	-0,47	0,033	Resident 3	0.155	-0,005	0,315	0.212	-0,04	0,464
	(0.077)			(0.123)				(0.089)			(0.13)				(0.081)			(0.131)		
Female	-0.371***	-0,561	-0,181	-0.257	-0,631	0,118	Female	-0.441***	-0,668	-0,21	-0.615*	-1,06	-0,17	Female	-0.138	-0,37	0,094	0.275	-0,085	0,635
	(0.097)			(0.194)				(0.116)			(0.226)				(0.119)			(0.182)		
ICC	0.9142			0.8474				0.9299			0.8602				0.9433			0.8874		
Observations	510			183				422			144				321			99		
Log Likelihood	-604.979			-197.576				-503.708			-155.368				-350.531			-81.525		
AIC	1239.958			425.151				1035.415			338.736				729.062			191.051		
BIC	1303.474			473.294				1092.045			380.313				781.862			227.382		

Note: B = Beta Coefficient, SE = Standard Error, CI = Confidence Interval, *p<0.05; **p<0.01, ***p<0.001, Benjamini-Hochberg correction for multiple comparisons

Table S.13 T-test results for professional efficacy: GP residents versus residents (full dataset)

T-Test	B (SE)	df	t.ratio	p.value
Year 1	-0.223 (0.136)	105	-1.635	0.126
Year 2	-0.220 (0.142)	105	-1.542	0.126
Year 3	-0.360 (0.135)	105	-2.672	0.026

Note: B = Beta Coefficient, SE = Standard Error, df = Degrees of Freedom, *Benjamini-Hochberg correction for multiple comparisons

Table S.14 Evolution of engagement in three cohorts (full and complete datasets)

Cohort 1	Full dataset			Complete dataset			Cohort 2	Full dataset			Complete dataset			Cohort 3	Full dataset			Complete dataset		
	B (SE)	CI (95%)		B (SE)	CI (95%)			B (SE)	CI (95%)		B (SE)	CI (95%)			B (SE)	CI (95%)		B (SE)	CI (95%)	
		Lower	Upper		Lower	Upper			Lower	Upper		Lower	Upper			Lower	Upper		Lower	Upper
(Intercept)	4.556***	4,324	4,788	4.686***	4,21	5,163	(Intercept)	3.848***	3,524	4,173	4.112***	3,515	4,709	(Intercept)	4.872***	4,587	5,156	4.593***	3,98	5,206
	(0.119)			(0.246)				(0.166)			(0.308)				(0.146)			(0.319)		
Bachelor 2	-0.05	-0,19	0,091	-0.06	-0,255	0,135	Master 2	-0.078	-0,238	0,081	0.03	-0,22	0,282	Resident 2	-0.34***	-0,523	-0,157	-0.219	-0,544	0,106
	(0.072)			(0.101)				(0.082)			(0.13)				(0.093)			(0.169)		
Bachelor 3	-0.276**	-0,427	-0,125	-0.315*	-0,532	-0,098	Master 3	-0.141	-0,31	0,029	0.058	-0,16	0,279	Resident 3	-0.423***	-0,631	-0,215	-0.296	-0,638	0,046
	(0.077)			(0.112)				(0.086)			(0.114)				(0.106)			(0.178)		
Female	-0.314*	-0,544	-0,085	-0.347	-0,81	0,117	Female	-0.265	-0,556	0,026	-0.656*	-1,27	-0,04	Female	-0.33*	-0,612	-0,047	-0.04	-0,684	0,604
	(0.117)			(0.239)				(0.149)			(0.313)				(0.145)			(0.326)		
ICC	0.9372			0.8882				0.94			0.8823				0.941			0.8685		
Observations	510			183				422			144				321			99		
Log Likelihood	-651.419			-206.664				-570.744			-171.259				-415.777			-121.619		
AIC	1332.838			443.327				1169.488			370.518				859.554			271.237		
BIC	1396.354			491.47				1226.118			412.096				912.354			307.569		

Note: B = Beta Coefficient, SE = Standard Error, CI = Confidence Interval, *p<0.05; **p<0.01, ***p<0.001, Benjamini-Hochberg correction for multiple comparisons

Table S.15 T-test results for engagement: GP residents versus residents (full dataset)

T-Test	B (SE)	df	t.ratio	p.value
Year 1	0.008 (0.170)	105	0.045	0.964
Year 2	0.159 (0.158)	105	1.008	0.518
Year 3	-0.182 (0.192)	105	-0.948	0.518

Note: B = Beta Coefficient, SE = Standard Error, df = Degrees of Freedom, *Benjamini-Hochberg correction for multiple comparisons

Table S.16 Determinants of emotional exhaustion across three cohorts (full and complete datasets)

Emotional exhaustion	Full dataset			Complete dataset		
Cohort 1	B (SE)	CI (95%)		B (SE)	CI (95%)	
		Lower	Upper		Lower	Upper
(Intercept)	2.915 (0.110)***	2.702	3.127	2.871 (0.219)***	2,458	3,283

Bachelor 2	0.079 (0.076)	-0,067	0,226	0.109 (0.113)	-0,104	0,322
Bachelor 3	0.105 (0.087)	-0,065	0,275	0.215 (0.141)	-0,051	0,48
Female	-0.191 (0.106)	-0,395	0,014	-0.409 (0.203)	-0,793	-0,025
Cognitive demands	0.001 (0.043)	-0,083	0,084	-0.022 (0.065)	-0,144	0,1
Workload	0.266 (0.046)***	0,176	0,356	0.225 (0.069)**	0,094	0,355
Work-Home conflict	0.513 (0.056)***	0,405	0,621	0.382 (0.081)***	0,229	0,536
Meaningfulness	0.009 (0.045)	-0,079	0,096	0.029 (0.074)	-0,111	0,17
Learning opportunities	-0.1 (0.044)	-0,185	-0,015	-0.125 (0.067)	-0,252	0,002
Learning environment	0.059 (0.042)	-0,023	0,142	0.096 (0.062)	-0,021	0,212
Neuroticism	0.332 (0.048)***	0,239	0,426	0.333 (0.079)***	0,184	0,482
Perfectionistic strivings	-0.037 (0.044)	-0,123	0,049	0.032 (0.074)	-0,108	0,172
Perfectionistic concerns	0.074 (0.046)	-0,016	0,164	0.24 (0.079)**	0,092	0,389
ICC		0.9203			0.8727	
Observations		510			183	
Log Likelihood		-608.748			-200.474	
AIC		1265.496			448.948	
BIC		1367.122			525.976	

Cohort 2	B (SE)	Full dataset	CI (95%)	B (SE)	Complete dataset	CI (95%)
		Lower	Upper		Lower	Upper
(Intercept)	2.715 (0.13)***	2,464	2,966	2.981 (0.193)***	2,619	3,343
Master 2	0.056 (0.091)	-0,121	0,233	-0.08 (0.126)	-0,317	0,157
Master 3	-0.004 (0.111)	-0,219	0,211	-0.136 (0.144)	-0,406	0,135
Female	0.092 (0.108)	-0,117	0,302	-0.266 (0.183)	-0,615	0,082
Cognitive demands	-0.036 (0.049)	-0,13	0,059	-0.075 (0.078)	-0,222	0,072
Workload	0.216 (0.051)***	0,117	0,315	0.119 (0.081)	-0,033	0,271
Work-Home conflict	0.573 (0.055)***	0,466	0,681	0.585 (0.093)***	0,41	0,76
Meaningfulness	-0.083 (0.049)	-0,178	0,012	-0.102 (0.075)	-0,242	0,038
Learning opportunities	-0.093 (0.05)	-0,189	0,004	0.025 (0.078)	-0,121	0,17
Learning environment	-0.032 (0.047)	-0,122	0,059	-0.102 (0.074)	-0,24	0,037
Neuroticism	0.324 (0.055)***	0,218	0,430	0.385 (0.094)***	0,21	0,561
Perfectionistic strivings	-0.103 (0.049)	-0,197	-0,009	-0.175 (0.077)	-0,319	-0,031
Perfectionistic concerns	0.122 (0.054)	0,017	0,227	0.116 (0.088)	-0,05	0,281
ICC		0.9158			0.8247	
Observations		422			144	
Log Likelihood		-503.343			-140.468	
AIC		1052.685			326.935	
BIC		1145.721			395.241	

Cohort 3	B (SE)	Full dataset	CI (95%)	B (SE)	Complete dataset	CI (95%)
		Lower	Upper		Lower	Upper
(Intercept)	2.851 (0.123)***	2,615	3,087	3.211 (0.281)***	2,695	3,727
Resident 2	0.043 (0.088)	-0,128	0,214	-0.201 (0.154)	-0,482	0,081
Resident 3	0.222 (0.1)	0,029	0,416	-0.058 (0.171)	-0,372	0,256
Female	-0.022 (0.118)	-0,249	0,204	-0.057 (0.3)	-0,619	0,505
Cognitive demands	-0.015 (0.046)	-0,105	0,074	-0.001 (0.085)	-0,157	0,155
Workload	0.148 (0.052)**	0,048	0,248	0.164 (0.089)	0,001	0,327
Work-Home conflict	0.575 (0.057)***	0,465	0,684	0.517 (0.1)***	0,335	0,7
Meaningfulness	-0.160 (0.052)**	-0,261	-0,058	-0.055 (0.096)	-0,231	0,122
Learning opportunities	-0.069 (0.044)	-0,154	0,015	-0.07 (0.071)	-0,200	0,06
Learning environment	-0.058 (0.043)	-0,141	0,025	-0.068 (0.07)	-0,197	0,061
Neuroticism	0.423 (0.057)***	0,312	0,533	0.464 (0.12)***	0,243	0,684
Perfectionistic strivings	-0.096 (0.054)	-0,200	0,007	-0.145 (0.107)	-0,341	0,05
Perfectionistic concerns	0.065 (0.056)	-0,043	0,173	0.166 (0.108)	-0,032	0,364
ICC		0.9193			0.855	
Observations		321			99	
Log Likelihood		-347.991			-99.854	
AIC		741.982			245.707	
BIC		828.725			305.395	

Note: B = Beta Coefficient, SE = Standard Error, CI = Confidence Interval, *p<0.05; **p<0.01,***p<0.001, Benjamini-Hochberg correction for multiple comparisons.

Table S.17 Cross-lagged panel analyses for emotional exhaustion across three cohorts (full dataset)

Cohort 1	Cross-lagged Panel Analysis T0 - T1			Cross-lagged Panel Analysis T1 - T2			
**Emotional exhaustion (T1)**	**B (SE)**	**CI (95%)**		**Emotional exhaustion (T2)**	**B (SE)**	**CI (95%)**	
		Lower	Upper			Lower	Upper
(Intercept)	2.942 (0.267)***	2,459	3,425	(Intercept)	3.08 (0.299)***	2,540	3,621

Cognitive demands (T0)	-0.016 (0.12)	-0,233	0,202	Cognitive demands (T1)	0.122 (0.133)	-0,118	0,362
Workload (T0)	0.298 (0.136)	0,051	0,544	Workload (T1)	-0.098 (0.147)	-0,365	0,168
Work-home conflict (T0)	0.23 (0.142)	-0,028	0,487	Work-home conflict (T1)	0.483 (0.165)*	0,185	0,782
Meaningfulness (T0)	0.151 (0.131)	-0,086	0,388	Meaningfulness (T1)	-0.058 (0.168)	-0,363	0,247
Learning opportunities (T0)	-0.256 (0.12)	-0,475	-0,038	Learning opportunities (T1)	-0.137 (0.176)	-0,455	0,181
Learning environment (T0)	0.03 (0.121)	-0,190	0,250	Learning environment (T1)	0.016 (0.128)	-0,216	0,248
Neuroticism (T0)	0.514 (0.146)**	0,249	0,779	Neuroticism (T1)	0.226 (0.136)	-0,02	0,473
Perfectionistic strivings (T0)	-0.036 (0.136)	-0,282	0,210	Perfectionistic strivings (T1)	-0.211 (0.152)	-0,486	0,064
Perfectionistic concerns (T0)	-0.039 (0.139)	-0,290	0,212	Perfectionistic concerns (T1)	0.426 (0.161)	0,133	0,718
ICC		0.8767		ICC		0.8767	
Observations		87		Observations		85	
Log Likelihood		-108.539		Log Likelihood		-116.788	
AIC		251.078		AIC		267.576	
BIC		292.998		BIC		309.101	

Cohort 2	Cross-lagged Panel Analysis T0 - T1				Cross-lagged Panel Analysis T1 - T2		
Emotional exhaustion (T1)	B (SE)	CI (95%)		Emotional exhaustion (T2)	B (SE)	CI (95%)	
		Lower	Upper			Lower	Upper
(Intercept)	2.736 (0.292)***	2,208	3,264	(Intercept)	3.352 (0.282)***	2,844	3,860
Cognitive demands (T0)	0.388 (0.149)*	0,118	0,658	Cognitive demands (T1)	0.01 (0.157)	-0,273	0,292
Workload (T0)	0.316 (0.162)	0,023	0,609	Workload (T1)	0.288 (0.142)*	0,033	0,543
Work-home conflict (T0)	0.011 (0.178)	-0,311	0,332	Work-home conflict (T1)	0.281 (0.169)	-0,023	0,585
Meaningfulness (T0)	-0.042 (0.152)	-0,318	0,233	Meaningfulness (T1)	-0.166 (0.129)	-0,398	0,066
Learning opportunities (T0)	0.094 (0.166)	-0,205	0,393	Learning opportunities (T1)	0.03 (0.156)	-0,251	0,311
Learning environment (T0)	0.038 (0.147)	-0,227	0,303	Learning environment (T1)	-0.058 (0.146)	-0,320	0,205
Neuroticism (T0)	0.552 (0.172)*	0,240	0,863	Neuroticism (T1)	0.189 (0.178)	-0,131	0,510
Perfectionistic strivings (T0)	-0.314 (0.158)	-0,599	-0,029	Perfectionistic strivings (T1)	-0.038 (0.13)	-0,271	0,195
Perfectionistic concerns (T0)	0.224 (0.161)	-0,067	0,515	Perfectionistic concerns (T1)	0.185 (0.165)	-0,111	0,482
ICC		0.8767		ICC		0.8767	
Observations		77		Observations		73	
Log Likelihood		-101.641		Log Likelihood		-92.455	
AIC		235.281		AIC		216.91	
BIC		272.782		BIC		253.558	

Cohort 3	Cross-lagged Panel Analysis T0 - T1				Cross-lagged Panel Analysis T1 - T2		
Emotional exhaustion (T1)	B (SE)	CI (95%)		Emotional exhaustion (T2)	B (SE)	CI (95%)	
		Lower	Upper			Lower	Upper
(Intercept)	2.995 (0.333)***	2,420	3,570	(Intercept)	2.768 (0.357)***	2,159	3,377
Cognitive demands (T0)	-0.132 (0.19)	-0,460	0,196	Cognitive demands (T1)	-0.239 (0.191)	-0,565	0,087
Workload (T0)	0.169 (0.186)	-0,152	0,490	Workload (T1)	-0.073 (0.208)	-0,428	0,282
Work-home conflict (T0)	0.695 (0.202)**	0,347	1,043	Work-home conflict (T1)	0.827 (0.253)*	0,396	1,258
Meaningfulness (T0)	0.23 (0.179)	-0,079	0,538	Meaningfulness (T1)	-0.393 (0.194)	-0,724	-0,061
Learning opportunities (T0)	-0.043 (0.174)	-0,343	0,256	Learning opportunities (T1)	0.074 (0.169)	-0,214	0,363
Learning environment (T0)	0.032 (0.146)	-0,219	0,283	Learning environment (T1)	0.141 (0.156)	-0,124	0,407
Neuroticism (T0)	0.524 (0.163)*	0,242	0,805	Neuroticism (T1)	-0.073 (0.226)	-0,458	0,313
Perfectionistic strivings (T0)	-0.102 (0.178)	-0,409	0,206	Perfectionistic strivings (T1)	0.020 (0.213)	-0,342	0,383
Perfectionistic concerns (T0)	0.162 (0.187)	-0,160	0,484	Perfectionistic concerns (T1)	0.164 (0.189)	-0,158	0,485
ICC		0.8767		ICC		0.8767	
Observations		51		Observations		47	
Log Likelihood		-61.218		Log Likelihood		-55.626	
AIC		154.437		AIC		143.252	
BIC		185.346		BIC		172.854	

Note: B = Beta Coefficient, SE = Standard Error, CI = Confidence Interval, *p<0.05; **p<0.01,***p<0.001, Benjamini-Hochberg correction for multiple comparisons

Table S.18 Determinants of cynicism across three cohorts (full and complete datasets)

Cynicism	Full dataset			Complete dataset		
Cohort 1	B (SE)	CI (95%)		B (SE)	CI (95%)	
		Lower	Upper		Lower	Upper
(Intercept)	1.219 (0.109)***	1,008	1,430	1.466 (0.226)***	1,04	1,893
Bachelor 2	0.09 (0.085)	-0,076	0,255	0.019 (0.152)	-0,267	0,306
Bachelor 3	0.253 (0.095)*	0,069	0,436	0.157 (0.169)	-0,162	0,476
Female	-0.139 (0.104)	-0,341	0,063	-0.444 (0.202)	-0,826	-0,063
Cognitive demands	-0.034 (0.046)	-0,123	0,055	-0.102 (0.079)	-0,25	0,047
Workload	0.218 (0.05)***	0,121	0,314	0.12 (0.086)	-0,042	0,283
Work-Home conflict	0.339 (0.059)***	0,226	0,453	0.366 (0.096)**	0,184	0,548
Meaningfulness	-0.221 (0.048)***	-0,314	-0,128	-0.253 (0.088)*	-0,42	-0,086
Learning opportunities	-0.135 (0.047)*	-0,226	-0,043	-0.042 (0.081)	-0,195	0,112
Learning environment	-0.029 (0.046)	-0,117	0,060	-0.025 (0.076)	-0,168	0,117

Neuroticism	0.144 (0.050)*	0,047	0,241	0.134 (0.087)	-0,031	0,298
Perfectionistic strivings	-0.089 (0.046)	-0,179	0,001	-0.191 (0.086)*	-0,353	-0,029
Perfectionistic concerns	0.062 (0.049)	-0,032	0,156	0.206 (0.093)*	0,031	0,381
ICC		0.9001			0.8596	
Observations		510			183	
Log Likelihood		-636.574			-227.03	
AIC		1321.149			502.06	
BIC		1422.775			579.088	

Cohort 2	Full dataset B (SE)	CI (95%)		Complete dataset B (SE)	CI (95%)	
		Lower	Upper		Lower	Upper
(Intercept)	1.483 (0.167)***	1,161	1,805	1.397 (0.268)***	0,893	1,900
Master 2	0.324 (0.099)**	0,131	0,516	0.194 (0.116)	-0,024	0,412
Master 3	0.287 (0.128)*	0,039	0,534	-0.014 (0.152)	-0,3	0,271
Female	-0.147 (0.147)	-0,431	0,137	0.16 (0.271)	-0,356	0,675
Cognitive demands	-0.004 (0.058)	-0,116	0,107	-0.07 (0.082)	-0,224	0,084
Workload	0.212 (0.063)**	0,089	0,335	0.076 (0.088)	-0,090	0,241
Work-Home conflict	0.298 (0.068)***	0,166	0,429	0.490 (0.098)***	0,306	0,674
Meaningfulness	-0.311 (0.059)***	-0,426	-0,197	-0.336 (0.08)***	-0,486	-0,185
Learning opportunities	-0.169 (0.059)*	-0,284	-0,054	-0.101 (0.081)	-0,253	0,052
Learning environment	-0.153 (0.057)*	-0,263	-0,044	-0.178 (0.08)	-0,329	-0,027
Neuroticism	0.227 (0.068)**	0,095	0,358	0.089 (0.104)	-0,106	0,284
Perfectionistic strivings	-0.059 (0.061)	-0,178	0,059	-0.153 (0.087)	-0,315	0,009
Perfectionistic concerns	0.046 (0.066)	-0,082	0,175	0.09à (0.096)	-0,090	0,270
ICC		0.9248			0.876	
Observations		422			144	
Log Likelihood		-589.875			-156.763	
AIC		1225.75			359.526	
BIC		1318.785			427.832	

Cohort 3	Full dataset B (SE)	CI (95%)		Complete dataset B (SE)	CI (95%)	
		Lower	Upper		Lower	Upper
(Intercept)	2.303 (0.157)***	2,000	2,605	2.002 (0.368)***	1,327	2,676
Resident 2	-0.101 (0.109)	-0,312	0,11	-0.031 (0.174)	-0,351	0,288
Resident 3	-0.011 (0.119)	-0,241	0,219	-0.248 (0.151)	-0,526	0,03
Female	-0.452 (0.152)*	-0,745	-0,16	-0.113 (0.372)	-0,81	0,584
Cognitive demands	-0.035 (0.058)	-0,148	0,077	0.248 (0.09)*	0,083	0,413
Workload	0.06 (0.064)	-0,065	0,185	-0.029 (0.086)	-0,187	0,129
Work-Home conflict	0.422 (0.070)***	0,286	0,559	0.251 (0.091)*	0,084	0,419
Meaningfulness	-0.237 (0.067)**	-0,368	-0,107	-0.272 (0.102)*	-0,459	-0,085
Learning opportunities	-0.075 (0.057)	-0,185	0,035	-0.037 (0.078)	-0,18	0,105
Learning environment	-0.019 (0.056)	-0,127	0,09	0.001 (0.081)	-0,149	0,15
Neuroticism	0.04 (0.073)	-0,101	0,182	0.161 (0.133)	-0,083	0,405
Perfectionistic strivings	-0.027 (0.069)	-0,161	0,107	0.026 (0.118)	-0,19	0,242
Perfectionistic concerns	0.167 (0.072)	0,028	0,306	-0.11 (0.122)	-0,335	0,114
ICC		0.9237			0.9226	
Observations		321			99	
Log Likelihood		-429.054			-111.17	
AIC		904.108			268.339	
BIC		990.851			328.027	

Note: B = Beta Coefficient, SE = Standard Error, CI = Confidence Interval, *p<0.05; **p<0.01,***p<0.001, Benjamini-Hochberg correction for multiple comparisons.

Table S.19 Cross-lagged panel analyses for cynicism across three cohorts (full dataset)

Cohort 1	Cross-lagged Panel Analysis T0 - T1			Cross-lagged Panel Analysis T1 - T2			
Cynicism (T1)	B (SE)	CI (95%)		Cynicism (T2)	B (SE)	CI (95%)	
		Lower	Upper			Lower	Upper
(Intercept)	1.208 (0.238)***	0,777	1,639	(Intercept)	1.7 (0.328)***	1,107	2,294
Cognitive demands (T0)	-0.066 (0.107)	-0,26	0,128	Cognitive demands (T1)	-0.113 (0.146)	-0,376	0,151
Workload (T0)	0.278 (0.121)	0,058	0,497	Workload (T1)	0.016 (0.162)	-0,276	0,309
Work-home conflict (T0)	0.155 (0.127)	-0,075	0,385	Work-home conflict (T1)	0.358 (0.181)	0,030	0,686
Meaningfulness (T0)	-0.177 (0.117)	-0,388	0,035	Meaningfulness (T1)	-0.095 (0.185)	-0,43	0,239
Learning opportunities (T0)	-0.032 (0.107)	-0,227	0,162	Learning opportunities (T1)	-0.188 (0.193)	-0,538	0,161
Learning environment (T0)	0.023 (0.108)	-0,173	0,219	Learning environment (T1)	0.001 (0.141)	-0,254	0,256
Neuroticism (T0)	0.243 (0.13)	0,007	0,48	Neuroticism (T1)	-0.004 (0.15)	-0,275	0,267
Perfectionistic strivings (T0)	-0.097 (0.121)	-0,316	0,122	Perfectionistic strivings (T1)	-0.518 (0.167)*	-0,821	-0,216
Perfectionistic concerns (T0)	0.088 (0.124)	-0,136	0,312	Perfectionistic concerns (T1)	0.523 (0.177)*	0,202	0,843
ICC		0.8767		ICC		0.8767	

Observations		87		Observations		85	
Log Likelihood		-98.583		Log Likelihood		-124.768	
AIC		231.167		AIC		283.536	
BIC		273.087		BIC		325.061	

**Cohort 2**	**Cross-lagged Panel Analysis T0 - T1**				**Cross-lagged Panel Analysis T1 - T2**		
**Cynicism (T1)**	**B (SE)**	**CI (95%)**		**Cynicism (T2)**	**B (SE)**	**CI (95%)**	
		**Lower**	**Upper**			**Lower**	**Upper**
(Intercept)	1.927 (0.322)***	1,345	2,509	(Intercept)	2.067 (0.391)***	1,365	2,77
Cognitive demands (T0)	0.297 (0.165)	0,000	0,595	Cognitive demands (T1)	-0.213 (0.217)	-0,604	0,178
Workload (T0)	0.357 (0.179)	0,034	0,681	Workload (T1)	0.285 (0.196)	-0,067	0,638
Work-home conflict (T0)	-0.130 (0.196)	-0,484	0,225	Work-home conflict (T1)	0.275 (0.234)	-0,146	0,696
Meaningfulness (T0)	-0.468 (0.168)*	-0,771	-0,164	Meaningfulness (T1)	-0.255 (0.179)	-0,577	0,066
Learning opportunities (T0)	0.099 (0.182)	-0,231	0,429	Learning opportunities (T1)	-0.027 (0.216)	-0,416	0,361
Learning environment (T0)	-0.075 (0.162)	-0,367	0,218	Learning environment (T1)	-0.271 (0.202)	-0,635	0,092
Neuroticism (T0)	0.530 (0.190)*	0,186	0,873	Neuroticism (T1)	-0.059 (0.247)	-0,503	0,385
Perfectionistic strivings (T0)	-0.139 (0.174)	-0,453	0,175	Perfectionistic strivings (T1)	0.223 (0.179)	-0,100	0,546
Perfectionistic concerns (T0)	0.13 (0.177)	-0,191	0,451	Perfectionistic concerns (T1)	0.176 (0.228)	-0,235	0,586
ICC		0.8767		ICC		0.8767	
Observations		77		Observations		73	
Log Likelihood		-109.141		Log Likelihood		-116.172	
AIC		250.282		AIC		264.345	
BIC		287.783		BIC		300.992	

**Cohort 3**	**Cross-lagged Panel Analysis T0 - T1**				**Cross-lagged Panel Analysis T1 - T2**		
**Cynicism (T1)**	**B (SE)**	**CI (95%)**		**Cynicism (T2)**	**B (SE)**	**CI (95%)**	
		**Lower**	**Upper**			**Lower**	**Upper**
(Intercept)	2.026 (0.428)***	1,287	2,765	(Intercept)	1.71 (0.359)***	1,098	2,322
Cognitive demands (T0)	-0.413 (0.245)	-0,835	0,009	Cognitive demands (T1)	-0.177 (0.192)	-0,504	0,151
Workload (T0)	0.193 (0.239)	-0,220	0,606	Workload (T1)	-0.118 (0.209)	-0,474	0,239
Work-home conflict (T0)	0.622 (0.259)	0,174	1,069	Work-home conflict (T1)	0.737 (0.254)*	0,304	1,170
Meaningfulness (T0)	0.153 (0.23)	-0,244	0,549	Meaningfulness (T1)	-0.256 (0.195)	-0,589	0,076
Learning opportunities (T0)	0.018 (0.223)	-0,367	0,403	Learning opportunities (T1)	-0.170 (0.170)	-0,460	0,119
Learning environment (T0)	-0.076 (0.187)	-0,399	0,246	Learning environment (T1)	-0.081 (0.156)	-0,348	0,185
Neuroticism (T0)	0.183 (0.21)	-0,178	0,545	Neuroticism (T1)	-0.479 (0.227)	-0,866	-0,093
Perfectionistic strivings (T0)	-0.443 (0.229)	-0,839	-0,048	Perfectionistic strivings (T1)	0.038 (0.214)	-0,326	0,402
Perfectionistic concerns (T0)	0.149 (0.24)	-0,265	0,563	Perfectionistic concerns (T1)	0.416 (0.189)	0,093	0,739
ICC		0.8767		ICC		0.8767	
Observations		51		Observations		47	
Log Likelihood		-74.04		Log Likelihood		-55.82	
AIC		180.081		AIC		143.641	
BIC		210.99		BIC		173.243	

Note: B = Beta Coefficient, SE = Standard Error, CI = Confidence Interval, *p<0.05; **p<0.01,***p<0.001, Benjamini-Hochberg correction for multiple comparisons

Table S.20 Determinants of professional efficacy across three cohorts (full and complete datasets)

**Professional efficacy**	**Full dataset**			**Complete dataset**		
**Cohort 1**	**B (SE)**	**CI (95%)**		**B (SE)**	**CI (95%)**	
		**Lower**	**Upper**		**Lower**	**Upper**
(Intercept)	3.89 (0.09)***	3,715	4,065	4.011 (0.183)***	3,666	4,356
Bachelor 2	-0.193 (0.071)*	-0,33	-0,055	-0.162 (0.095)	-0,342	0,018
Bachelor 3	-0.347 (0.078)***	-0,498	-0,195	-0.372 (0.133)*	-0,623	-0,121
Female	-0.211 (0.085)*	-0,376	-0,045	-0.14 (0.175)	-0,471	0,19
Cognitive demands	0.032 (0.038)	-0,043	0,106	0.056 (0.061)	-0,059	0,171
Workload	-0.178 (0.042)***	-0,258	-0,097	-0.137 (0.066)	-0,261	-0,013
Work-Home conflict	0.049 (0.049)	-0,046	0,143	0.09 (0.073)	-0,048	0,229
Meaningfulness	0.118 (0.040)**	0,041	0,195	0.024 (0.067)	-0,103	0,151
Learning opportunities	0.142 (0.039)**	0,066	0,218	0.046 (0.062)	-0,07	0,162
Learning environment	0.036 (0.038)	-0,037	0,110	0.170 (0.059)*	0,06	0,281
Neuroticism	-0.198 (0.042)***	-0,279	-0,117	-0.195 (0.073)*	-0,333	-0,057
Perfectionistic strivings	0.157 (0.038)***	0,083	0,232	0.134 (0.067)	0,008	0,26
Perfectionistic concerns	-0.034 (0.04)	-0,113	0,044	-0.034 (0.075)	-0,175	0,107
ICC		0.9037			0.8409	
Observations		510			183	
Log Likelihood		-542.542			-181.31	
AIC		1133.084			410.621	
BIC		1234.71			487.648	

	**Full dataset**			**Complete dataset**		
**Cohort 2**	**B (SE)**	**CI (95%)**		**B (SE)**	**CI (95%)**	
		**Lower**	**Upper**		**Lower**	**Upper**

(Intercept)	4.054 (0.102)***	3,857	4,252	3.984 (0.17)***	3,664	4,303
Master 2	-0.386 (0.067)***	-0,517	-0,255	-0.317 (0.113)*	-0,529	-0,104
Master 3	-0.432 (0.080)***	-0,587	-0,278	-0.316 (0.105)*	-0,513	-0,119
Female	-0.255 (0.090)*	-0,429	-0,081	-0.232 (0.17)	-0,557	0,092
Cognitive demands	0.027 (0.039)	-0,049	0,102	0.036 (0.067)	-0,089	0,161
Workload	-0.205 (0.042)***	-0,286	-0,125	-0.184 (0.07)*	-0,316	-0,052
Work-Home conflict	0.099 (0.045)*	0,012	0,185	0.074 (0.08)	-0,076	0,225
Meaningfulness	0.104 (0.039)*	0,028	0,179	0.035 (0.063)	-0,083	0,152
Learning opportunities	0.236 (0.039)***	0,160	0,311	0.253 (0.063)**	0,135	0,372
Learning environment	0.104 (0.038)*	0,032	0,177	0.201 (0.061)**	0,086	0,316
Neuroticism	-0.282 (0.044)***	-0,368	-0,196	-0.206 (0.081)*	-0,359	-0,054
Perfectionistic strivings	0.144 (0.040)***	0,067	0,221	0.167 (0.069)*	0,038	0,296
Perfectionistic concerns	-0.091 (0.044)*	-0,175	-0,006	-0.188 (0.079)*	-0,336	-0,04
ICC		0.9027			0.8006	
Observations		422			144	
Log Likelihood		-407.905			-118.116	
AIC		861.809			282.231	
BIC		954.844			350.537	

Cohort 3	Full dataset			Complete dataset		
	B (SE)	CI (95%)		B (SE)	CI (95%)	
		Lower	Upper		Lower	Upper
(Intercept)	4.933 (0.114)***	4,713	5,153	4.720 (0.173)***	4,402	5,037
Resident 2	-0.031 (0.078)	-0,182	0,12	0.016 (0.13)	-0,222	0,253
Resident 3	0.218 (0.081)*	0,061	0,375	0.261 (0.129)	0,024	0,497
Female	0 (0.115)	-0,22	0,22	0.454 (0.17)	0,135	0,773
Cognitive demands	0.019 (0.042)	-0,062	0,099	0.028 (0.056)	-0,075	0,132
Workload	0 (0.046)	-0,088	0,089	0.005 (0.065)	-0,114	0,124
Work-Home conflict	-0.078 (0.051)	-0,176	0,021	-0.086 (0.067)	-0,21	0,038
Meaningfulness	0.202 (0.047)***	0,110	0,293	0.204 (0.073)	0,07	0,338
Learning opportunities	0.085 (0.039)	0,009	0,16	0.056 (0.054)	-0,043	0,156
Learning environment	-0.012 (0.038)	-0,085	0,062	-0.008 (0.051)	-0,102	0,087
Neuroticism	-0.041 (0.053)	-0,143	0,06	-0.047 (0.077)	-0,188	0,094
Perfectionistic strivings	-0.09 (0.05)	-0,186	0,006	-0.104 (0.067)	-0,227	0,018
Perfectionistic concerns	-0.054 (0.051)	-0,153	0,045	-0.078 (0.071)	-0,209	0,052
ICC		0.9271			0.8574	
Observations		321			99	
Log Likelihood		-321.532			-67.393	
AIC		689.064			180.786	
BIC		775.807			240.474	

Note: B = Beta Coefficient, SE = Standard Error, CI = Confidence Interval, *p<0.05; **p<0.01,***p<0.001, Benjamini-Hochberg correction for multiple comparisons.

Table S.21 Cross-lagged panel analyses for professional efficacy across three cohorts (full dataset)

Cohort 1	Cross-lagged Panel Analysis T0 - T1			Cross-lagged Panel Analysis T1 - T2			
**Professional efficacy (T1)**	**B (SE)**	**CI (95%)**		**Professional efficacy (T2)**	**B (SE)**	**CI (95%)**	
		Lower	Upper			Lower	Upper
(Intercept)	3.852 (0.231)***	3,432	4,271	(Intercept)	3.598 (0.217)***	3,206	3,991
Cognitive demands (T0)	0.014 (0.104)	-0,175	0,203	Cognitive demands (T1)	-0.102 (0.096)	-0,276	0,072
Workload (T0)	-0.226 (0.118)	-0,440	-0,013	Workload (T1)	-0.087 (0.107)	-0,281	0,106
Work-home conflict (T0)	0.189 (0.123)	-0,035	0,413	Work-home conflict (T1)	0.079 (0.12)	-0,138	0,296
Meaningfulness (T0)	0.059 (0.114)	-0,147	0,265	Meaningfulness (T1)	0.035 (0.122)	-0,186	0,257
Learning opportunities (T0)	0.260 (0.105)	0,071	0,45	Learning opportunities (T1)	0.328 (0.128)	0,097	0,56
Learning environment (T0)	0.077 (0.105)	-0,114	0,268	Learning environment (T1)	-0.08 (0.093)	-0,248	0,089
Neuroticism (T0)	-0.196 (0.127)	-0,426	0,035	Neuroticism (T1)	-0.063 (0.099)	-0,242	0,116
Perfectionistic strivings (T0)	0.171 (0.118)	-0,043	0,385	Perfectionistic strivings (T1)	0.129 (0.111)	-0,071	0,33
Perfectionistic concerns (T0)	-0.043 (0.12)	-0,261	0,175	Perfectionistic concerns (T1)	0.015 (0.117)	-0,197	0,227
ICC		0.8767		ICC		0.8767	
Observations		87		Observations		85	
Log Likelihood		-96.258		Log Likelihood		-89.64	
AIC		226.516		AIC		213.279	
BIC		268.436		BIC		254.804	

Cohort 2	Cross-lagged Panel Analysis T0 - T1			Cross-lagged Panel Analysis T1 - T2			
**Professional efficacy (T1)**	**B (SE)**	**CI (95%)**		**Professional efficacy (T2)**	**B (SE)**	**CI (95%)**	
		Lower	Upper			Lower	Upper
(Intercept)	3.526 (0.206)***	3,153	3,899	(Intercept)	3.776 (0.186)***	3,441	4,111
Cognitive demands (T0)	-0.211 (0.105)*	-0,401	-0,02	Cognitive demands (T1)	0.105 (0.104)	-0,081	0,291
Workload (T0)	-0.343 (0.115)*	-0,55	-0,135	Workload (T1)	-0.261 (0.093)*	-0,429	-0,093

Work-home conflict (T0)	0.228 (0.126)	0,001	0,456	Work-home conflict (T1)	0.095 (0.111)	-0,106	0,295
Meaningfulness (T0)	0.035 (0.108)	-0,160	0,229	Meaningfulness (T1)	-0.042 (0.085)	-0,196	0,111
Learning opportunities (T0)	0.185 (0.117)	-0,026	0,396	Learning opportunities (T1)	0.413 (0.103)**	0,227	0,598
Learning environment (T0)	0.003 (0.104)	-0,185	0,19	Learning environment (T1)	-0.044 (0.096)	-0,217	0,129
Neuroticism (T0)	-0.429 (0.122)**	-0,649	-0,209	Neuroticism (T1)	-0.154 (0.117)	-0,365	0,058
Perfectionistic strivings (T0)	0.038 (0.111)	-0,164	0,239	Perfectionistic strivings (T1)	-0.013 (0.085)	-0,167	0,14
Perfectionistic concerns (T0)	-0.036 (0.114)	-0,241	0,17	Perfectionistic concerns (T1)	-0.035 (0.109)	-0,231	0,16
ICC		0.8767		ICC		0.8767	
Observations		77		Observations		73	
Log Likelihood		-74.878		Log Likelihood		-62.056	
AIC		181.756		AIC		156.113	
BIC		219.257		BIC		192.76	

**Cohort 3**	**Cross-lagged Panel Analysis T0 - T1**				**Cross-lagged Panel Analysis T1 - T2**		
**Professional efficacy (T1)**	**B (SE)**	**CI (95%)**		**Professional efficacy (T2)**	**B (SE)**	**CI (95%)**	
		**Lower**	**Upper**			**Lower**	**Upper**
(Intercept)	4.870 (0.28)***	4,388	5,353	(Intercept)	5.277 (0.213)***	4,914	5,64
Cognitive demands (T0)	0.355 (0.16)*	0,080	0,631	Cognitive demands (T1)	0.141 (0.114)	-0,053	0,335
Workload (T0)	-0.132 (0.156)	-0,402	0,137	Workload (T1)	0.084 (0.124)	-0,128	0,296
Work-home conflict (T0)	-0.208 (0.169)	-0,500	0,084	Work-home conflict (T1)	-0.369 (0.151)	-0,626	-0,113
Meaningfulness (T0)	0.284 (0.15)	0,025	0,543	Meaningfulness (T1)	0.114 (0.116)	-0,083	0,311
Learning opportunities (T0)	0.048 (0.146)	-0,203	0,299	Learning opportunities (T1)	0.007 (0.101)	-0,165	0,178
Learning environment (T0)	0.000 (0.122)	-0,211	0,211	Learning environment (T1)	0.047 (0.093)	-0,112	0,205
Neuroticism (T0)	-0.083 (0.137)	-0,319	0,153	Neuroticism (T1)	0.216 (0.135)	-0,013	0,446
Perfectionistic strivings (T0)	0.351 (0.15)	0,092	0,609	Perfectionistic strivings (T1)	0.064 (0.127)	-0,152	0,28
Perfectionistic concerns (T0)	-0.271 (0.157)	-0,541	-0,001	Perfectionistic concerns (T1)	-0.157 (0.112)	-0,348	0,035
ICC		0.8767		ICC		0.8767	
Observations		51		Observations		47	
Log Likelihood		-52.287		Log Likelihood		-31.273	
AIC		136.573		AIC		94.546	
BIC		167.483		BIC		124.148	

Note: B = Beta Coefficient, SE = Standard Error, CI = Confidence Interval, *p<0.05; **p<0.01,***p<0.001, Benjamini-Hochberg correction for multiple comparisons

Table S.22 Determinants of engagement across three cohorts (full dataset)

**Engagement**	**Full dataset**			**Complete dataset**		
**Cohort 1**	**B (SE)**	**CI (95%)**		**B (SE)**	**CI (95%)**	
		**Lower**	**Upper**		**Lower**	**Upper**
(Intercept)	4.427 (0.107)***	4,219	4,634	4.482 (0.226)***	4,055	4,909
Bachelor 2	-0.100 (0.075)	-0,245	0,045	0.005 (0.107)	-0,196	0,206
Bachelor 3	-0.27 (0.081)**	-0,426	-0,113	-0.196 (0.127)	-0,436	0,044
Female	-0.188 (0.103)	-0,387	0,012	-0.218 (0.214)	-0,622	0,185
Cognitive demands	0.082 (0.042)	0,001	0,163	0.063 (0.063)	-0,057	0,182
Workload	-0.141 (0.045)**	-0,228	-0,054	-0.117 (0.067)	-0,244	0,009
Work-Home conflict	-0.024 (0.054)	-0,129	0,080	0.096 (0.079)	-0,053	0,245
Meaningfulness	0.125 (0.043)**	0,041	0,209	0.082 (0.069)	-0,05	0,213
Learning opportunities	0.160 (0.042)**	0,078	0,242	0.059 (0.063)	-0,059	0,177
Learning environment	0.024 (0.041)	-0,055	0,104	0.109 (0.06)	-0,005	0,222
Neuroticism	-0.171 (0.047)**	-0,263	-0,079	-0.194 (0.08)	-0,344	-0,044
Perfectionistic strivings	0.215 (0.043)***	0,132	0,297	0.226 (0.069)*	0,095	0,357
Perfectionistic concerns	0.046 (0.045)	-0,042	0,133	0.002 (0.077)	-0,143	0,147
ICC		0.9249			0.8873	
Observations		510			183	
Log Likelihood		-593.227			-190.144	
AIC		1234.455			428.288	
BIC		1336.081			505.316	

	**Full dataset**			**Complete dataset**		
**Cohort 2**	**B (SE)**	**CI (95%)**		**B (SE)**	**CI (95%)**	
		**Lower**	**Upper**		**Lower**	**Upper**
(Intercept)	3.91 (0.136)***	3,646	4,173	4.216 (0.264)***	3,722	4,71
Master 2	-0.054 (0.077)	-0,203	0,095	0.05 (0.126)	-0,187	0,287
Master 3	-0.114 (0.081)	-0,271	0,043	0.052 (0.114)	-0,161	0,266
Female	-0.151 (0.125)	-0,392	0,089	-0.517 (0.271)	-1,032	-0,001
Cognitive demands	0.050 (0.044)	-0,036	0,135	0.021 (0.077)	-0,122	0,165
Workload	-0.124 (0.049)*	-0,220	-0,029	-0.203 (0.084)	-0,36	-0,046
Work-Home conflict	-0.057 (0.053)	-0,159	0,045	-0.078 (0.092)	-0,251	0,095
Meaningfulness	0.227 (0.044)***	0,141	0,312	0.179 (0.073)	0,042	0,315
Learning opportunities	0.187 (0.044)***	0,101	0,273	0.183 (0.072)	0,048	0,317

Learning environment	0.045 (0.043)	-0,038	0,128	-0.005 (0.07)	-0,137	0,127
Neuroticism	-0.242 (0.053)***	-0,346	-0,139	-0.097 (0.1)	-0,284	0,09
Perfectionistic strivings	0.174 (0.049)**	0,078	0,269	0.127 (0.083)	-0,027	0,282
Perfectionistic concerns	-0.029 (0.052)	-0,130	0,072	-0.047 (0.096)	-0,227	0,132
ICC		0.9304			0.8696	
Observations		422			144	
Log Likelihood		-490.194			-146.557	
AIC		1026.389			339.114	
BIC		1119.424			407.42	

	Full dataset			Complete dataset		
**Cohort 3**	**B (SE)**	**CI (95%)**		**B (SE)**	**CI (95%)**	
		**Lower**	**Upper**		**Lower**	**Upper**
(Intercept)	4.507 (0.119)***	4,279	4,735	4.269 (0.242)***	3,825	4,712
Resident 2	-0.119 (0.079)	-0,272	0,034	0.17 (0.143)	-0,093	0,433
Resident 3	-0.252 (0.095)*	-0,435	-0,069	-0.039 (0.174)	-0,358	0,28
Female	-0.053 (0.115)	-0,274	0,169	0.012 (0.239)	-0,438	0,461
Cognitive demands	0.061 (0.044)	-0,023	0,146	0.086 (0.079)	-0,059	0,231
Workload	-0.004 (0.049)	-0,098	0,090	-0.005 (0.082)	-0,156	0,146
Work-Home conflict	-0.206 (0.054)***	-0,309	-0,102	-0.212 (0.092)	-0,381	-0,043
Meaningfulness	0.328 (0.050)***	0,232	0,425	0.364 (0.088)***	0,203	0,525
Learning opportunities	0.135 (0.042)**	0,055	0,215	0.192 (0.067)*	0,069	0,316
Learning environment	0.068 (0.041)	-0,01	0,147	0.123 (0.065)	0,004	0,242
Neuroticism	-0.217 (0.055)***	-0,324	-0,109	-0.305 (0.107)*	-0,502	-0,107
Perfectionistic strivings	0.141 (0.052)*	0,041	0,241	0.167 (0.096)	-0,009	0,343
Perfectionistic concerns	-0.028 (0.054)	-0,131	0,076	0.022 (0.097)	-0,157	0,20
ICC		0.9245			0.8682	
Observations		321			99	
Log Likelihood		-333.934			-92.098	
AIC		713.867			230.195	
BIC		800.61			289.883	

Note: B = Beta Coefficient, SE = Standard Error, CI = Confidence Interval, *p<0.05; **p<0.01,***p<0.001, Benjamini-Hochberg correction for multiple comparisons.

Table S.23 Cross-lagged panel analyses for engagement for all cohorts (full datasets)

**Cohort 1**	**Cross-lagged Panel Analysis T0 - T1**				**Cross-lagged Panel Analysis T1 - T2**		
**Engagement (T1)**	**B (SE)**	**CI (95%)**		**Engagement (T2)**	**B (SE)**	**CI (95%)**	
		**Lower**	**Upper**			**Lower**	**Upper**
(Intercept)	4.434 (0.217)***	4,04	4,827	(Intercept)	4.185 (0.26)***	3,715	4,655
Cognitive demands (T0)	0.177 (0.098)	0,000	0,354	Cognitive demands (T1)	-0.019 (0.115)	-0,228	0,189
Workload (T0)	-0.138 (0.111)	-0,338	0,063	Workload (T1)	-0.059 (0.128)	-0,291	0,173
Work-home conflict (T0)	-0.065 (0.116)	-0,275	0,145	Work-home conflict (T1)	0.251 (0.144)	-0,009	0,511
Meaningfulness (T0)	0.056 (0.107)	-0,137	0,249	Meaningfulness (T1)	0.077 (0.146)	-0,188	0,342
Learning opportunities (T0)	0.157 (0.098)	-0,021	0,335	Learning opportunities (T1)	0.21 (0.153)	-0,067	0,486
Learning environment (T0)	-0.152 (0.099)	-0,331	0,027	Learning environment (T1)	-0.189 (0.111)	-0,391	0,013
Neuroticism (T0)	-0.238 (0.119)	-0,454	-0,022	Neuroticism (T1)	-0.052 (0.119)	-0,267	0,163
Perfectionistic strivings (T0)	0.278 (0.110)	0,078	0,478	Perfectionistic strivings (T1)	0.445 (0.132)*	0,205	0,684
Perfectionistic concerns (T0)	0.046 (0.113)	-0,159	0,251	Perfectionistic concerns (T1)	-0.221 (0.14)	-0,475	0,033
ICC		0.8767		ICC		0.8767	
Observations		87		Observations		85	
Log Likelihood		-90.684		Log Likelihood		-104.924	
AIC		215.367		AIC		243.848	
BIC		257.287		BIC		285.373	

**Cohort 2**	**Cross-lagged Panel Analysis T0 - T1**				**Cross-lagged Panel Analysis T1 - T2**		
**Engagement (T1)**	**B (SE)**	**CI (95%)**		**Engagement (T2)**	**B (SE)**	**CI (95%)**	
		**Lower**	**Upper**			**Lower**	**Upper**
(Intercept)	3.766 (0.289)***	3,245	4,288	(Intercept)	3.884 (0.284)***	3,374	4,395
Cognitive demands (T0)	0.083 (0.148)	-0,184	0,349	Cognitive demands (T1)	0.003 (0.158)	-0,281	0,287
Workload (T0)	-0.137 (0.16)	-0,427	0,153	Workload (T1)	-0.307 (0.142)	-0,563	-0,051
Work-home conflict (T0)	0.161 (0.176)	-0,157	0,479	Work-home conflict (T1)	0.105 (0.17)	-0,2	0,411
Meaningfulness (T0)	0.224 (0.151)	-0,048	0,497	Meaningfulness (T1)	0.026 (0.13)	-0,207	0,26
Learning opportunities (T0)	0.096 (0.164)	-0,2	0,392	Learning opportunities (T1)	0.442 (0.157)*	0,159	0,724
Learning environment (T0)	0.042 (0.145)	-0,22	0,304	Learning environment (T1)	0.074 (0.147)	-0,19	0,338
Neuroticism (T0)	-0.451 (0.17)	-0,759	-0,143	Neuroticism (T1)	-0.154 (0.179)	-0,477	0,168
Perfectionistic strivings (T0)	0.023 (0.156)	-0,259	0,305	Perfectionistic strivings (T1)	-0.009 (0.13)	-0,244	0,225
Perfectionistic concerns (T0)	0.04 (0.159)	-0,248	0,328	Perfectionistic concerns (T1)	0.112 (0.166)	-0,186	0,41
ICC		0.8767		ICC		0.8767	

Observations		77		Observations		73	
Log Likelihood		-100.754		Log Likelihood		-92.866	
AIC		233.507		AIC		217.732	
BIC		271.008		BIC		254.379	

**Cohort 3**	**Cross-lagged Panel Analysis T0 - T1**				**Cross-lagged Panel Analysis T1 - T2**		
**Engagement (T1)**	**B (SE)**	**CI (95%)**		**Engagement (T2)**	**B (SE)**	**CI (95%)**	
		**Lower**	**Upper**			**Lower**	**Upper**
(Intercept)	4.428 (0.32)***	3,876	4,98	(Intercept)	4.165 (0.337)***	3,59	4,739
Cognitive demands (T0)	0.242 (0.183)	-0,073	0,557	Cognitive demands (T1)	-0.006 (0.18)	-0,314	0,301
Workload (T0)	-0.102 (0.179)	-0,41	0,207	Workload (T1)	-0.157 (0.196)	-0,492	0,178
Work-home conflict (T0)	-0.321 (0.194)	-0,655	0,013	Work-home conflict (T1)	-0.079 (0.238)	-0,485	0,327
Meaningfulness (T0)	0.101 (0.172)	-0,195	0,398	Meaningfulness (T1)	0.369 (0.183)	0,056	0,681
Learning opportunities (T0)	0.03 (0.167)	-0,258	0,317	Learning opportunities (T1)	0.245 (0.159)	-0,027	0,516
Learning environment (T0)	-0.125 (0.14)	-0,366	0,116	Learning environment (T1)	-0.064 (0.147)	-0,314	0,187
Neuroticism (T0)	-0.329 (0.157)	-0,599	-0,059	Neuroticism (T1)	-0.247 (0.213)	-0,611	0,116
Perfectionistic strivings (T0)	0.435 (0.171)	0,14	0,73	Perfectionistic strivings (T1)	-0.037 (0.2)	-0,379	0,305
Perfectionistic concerns (T0)	-0.167 (0.179)	-0,476	0,142	Perfectionistic concerns (T1)	0.184 (0.178)	-0,119	0,487
ICC		0.8767		ICC		0.8767	
Observations		51		Observations		47	
Log Likelihood		-59.127		Log Likelihood		-52.852	
AIC		150.254		AIC		137.704	
BIC		181.164		BIC		167.306	

Note: B = Beta Coefficient, SE = Standard Error, CI = Confidence Interval, *p<0.05; **p<0.01,***p<0.001, Benjamini-Hochberg correction for multiple comparisons

Supplementary Figures

Dgespc Q,/ Npcbgarcb k c_l q n_rrcpl q dmp ck mgml _j cvf _sqrgml &dsjj b_r_qcr',

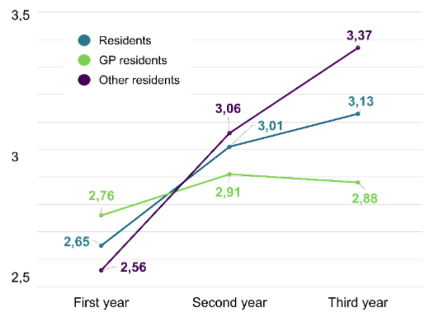

Dgespc Q,0 Npcbgarcb k c_l q n_rrcpl q dmp awl gagqk &dsjj b_r_qcr'

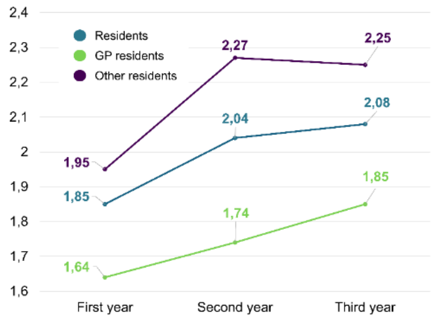

Dgespc Q,1 Npcbgarcb k c_l q n_rrcpl q dmp npmdcqqgml _j cdda_aw &dsjj b_r_qcr'

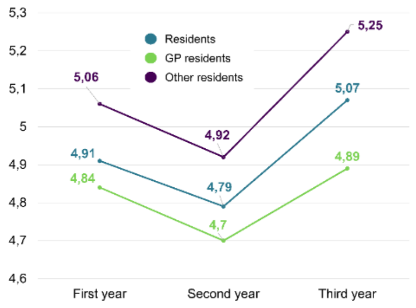

Dgespc Q,2 Npcbgarcb k c_l q md cl e_eck cl r &dsjj b_r_qcr'

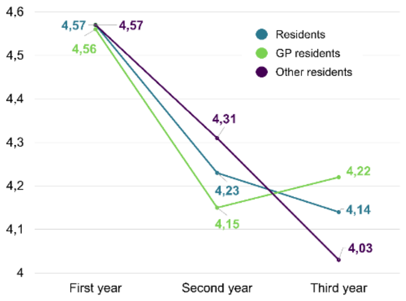

Supplement: Supplementary File. — Supplementary Tables S.1 to S.23 and Figures S.1 to S.4. [file pme-15-1-2013-s1.pdf]
